# Dataset of manually measured QT intervals in the electrocardiogram

**DOI:** 10.1186/1475-925X-5-31

**Published:** 2006-05-18

**Authors:** Ivaylo Christov, Ivan Dotsinsky, Iana Simova, Rada Prokopova, Elina Trendafilova, Stefan Naydenov

**Affiliations:** 1 Centre of Biomedical Engineering, Bulgarian Academy of Sciences, Acad. G. Bonchev str., block 105, 1113 Sofia, Bulgaria; 2 University Hospital "Aleksandrovska", Clinic of Cardiology, Sofia, Bulgaria; 3 University Hospital "St. Anna", Clinic of Internal Diseases, Sofia, Bulgaria; 4 National Heart Hospital, Coronary Care Unit, Sofia, Bulgaria; 5 University Hospital, Department of Internal Medicine "Prof. St. Kirkovic', Sofia, Bulgaria

## Abstract

**Background:**

The QT interval and the QT dispersion are currently a subject of considerable interest. Cardiac repolarization delay is known to favor the development of arrhythmias. The QT dispersion, defined as the difference between the longest and the shortest QT intervals or as the standard deviation of the QT duration in the 12-lead ECG is assumed to be reliable predictor of cardiovascular mortality.

The seventh annual PhysioNet/Computers in Cardiology Challenge, 2006 addresses a question of high clinical interest: **Can the QT interval be measured by fully automated methods with accuracy acceptable for clinical evaluations?**

**Method:**

The PTB Diagnostic ECG Database was given to 4 cardiologists and 1 biomedical engineer for manual marking of QRS onsets and T-wave ends in 458 recordings. Each recording consisted of one selected beat in lead II, chosen visually to have minimum baseline shift, noise, and artifact.

In cases where no T wave could be observed or its amplitude was very small, the referees were instructed to mark a 'group-T-wave end' taking into consideration leads with better manifested T wave.

A modified Delphi approach was used, which included up to three rounds of measurements to obtain results closer to the median.

**Results:**

A total amount of 2*5*548 Q-onsets and T-wave ends were manually marked during round 1. To obtain closer to the median results, 8.58 % of Q-onsets and 3.21 % of the T-wave ends had to be reviewed during round 2, and 1.50 % Q-onsets and 1.17 % T-wave ends in round 3.

The mean and standard deviation of the differences between the values of the referees and the median after round 3 were 2.43 ± 0.96 ms for the Q-onset, and 7.43 ± 3.44 ms for the T-wave end.

**Conclusion:**

A fully accessible, on the Internet, dataset of manually measured Q-onsets and T-wave ends was created and presented in additional file: 1 (Table 4) with this article. Thus, an available standard can be used for the development of automated methods for the detection of Q-onsets, T-wave ends and for QT interval measurements.

## Background

The QT interval in the electrocardiogram (ECG) represents the duration of ventricular depolarization and subsequent repolarization. It is measured from the beginning of the QRS complex to the end of the T wave. The 'group-Q-onset' and a 'group-T-wave end' are defined, respectively, as the earliest Q-onset and the latest T-wave end in a group of leads. The QT interval has always been of particular interest and is affected by many factors: heart rate, autonomic nervous tone, sympathomimetics, electrolytes especially calcium, some drugs, age, sex of the patient, and even sleep. An undesirable property of some antiarrhythmic drugs is their ability to delay cardiac repolarization, an effect that can be measured on the surface electrocardiogram as prolongation of the QT interval assessed against its initial width or compared to common accepted thresholds [1]. Cardiac repolarization delay is known to create an electrophysiological environment that favors the development of cardiac arrhythmias, most clearly torsade de pointes (TdP), but possibly other ventricular tachyarrhythmias as well. TdP is a polymorphic ventricular tachyarrhythmia that appears on the ECG as a continuous twisting of the vector of the QRS complex around the isoelectric baseline. A feature of TdP is a pronounced prolongation of the QT interval in the supraventricular beats preceding the arrhythmia. TdP can degenerate into ventricular fibrillation, leading to sudden death.

The QT dispersion defined as the difference between the longest and the shortest QT intervals or as the standard deviation of the QT duration in the 12-lead ECG [2], currently is the subject of significant interest. Several years ago, Campbell [3] enthusiastically called it the 'electrophysiological Holy Grail'. The number of studies indexed in Medline on QT dispersion is more than 1200 since its description in 1990.

Some authors are poles apart in their views on the QT measurement, because of the complexities of its assessment and interpretation. Examining the QT dispersion for predictability of cardiovascular mortality, Shah *et al*. [4] assumed that discrepancies among the studies may be explained by the difficulty in obtaining accurate and reproducible measures of QT intervals. Malik and Batchvarov [5] also paid attention to methodological difficulties in the measurement of the QT interval. These authors asserted that: (i) the reliability of both automatic and manual measurement of QT dispersion is low and significantly lower than that of the QT interval; (ii) the agreement between manual and automatic measurement is poor; (iii) the QT dispersion results mainly from variations in the T wave loop morphology and the error of the QT measurement.

The 'classical' problem of quantitative electrocardiography has been approached by the Common Standards of Electrocardiography (CSE) Working Party [6,7]. An international project consisting of active participants from 20 institutions of the European Community was initiated to overcome the lack of standards, to provide agreement on wave definitions, and to insure equality of measuring protocol [7]. A reference library was thereby established through a comprehensive interactive review process that was carried out by cardiologists on highly amplified ECG tracings. The CSE Working Group used repeated assessments in four rounds: the first three to correct the inter-observer differences and the fourth to correct the common referee's median with respect to program derived median.

Inter-observer measurements have in some cases been shown to vary considerably [8-10]. Computer programs tested against the CSE Working Group database [7,11,12] or another reference database [13-15] had similar differences.

Several works by Marray *et al*. are devoted to errors in the manual measurement of the QT intervals [16-18]. The authors have shown that longer QT intervals are reported by the experts with an increase of the amplification gain (8 ms for doubling the gain) and slower paper speed (11 ms going from 100 mm/s to 50 mm/s) [16]. The greatest mean difference reported of the Q-onset among four cardiologists was 6.7 ms at a gain of 5 mm/mV, which decreased to 3.2 ms at a gain of 10 mm/mV [17]. Faber *et al*. [19] claimed that the paper speed, but not the amplifier gain, has more effect on manual QT measurement.

Laguna *et al*. [20] presented a manually developed database to be used as a reference when validating wave boundary detectors. The database had 105 15-minute excerpts of two-channel ECGs. Within each record, between 30 and 100 representative beats were manually annotated by a single cardiologist. Just 11 ECGs were marked by 2 cardiologists, with the standard deviation of the T-wave end between the experts found to be 25.5 ms [10].

QT changes in successive beats have major clinical significance, but in our opinion software algorithms are better tested with the many shape, magnitude and slew-rate variety of the QRS and T waves. Successive beat detection is not of concern to software algorithms because the same detection (no matter if it is good or bad) will occur in more than 95 % of the successive beats having unchanged form and size, and assuming that no artifacts occur.

The 7th annual PhysioNet/Computers in Cardiology Challenge, 2006 [21] addressed a question of high clinical interest: **Can the QT interval be measured by fully automated methods with accuracy acceptable for clinical evaluations? **The data to be used for the challenge are the 549 recordings of the PTB Diagnostic ECG Database, which was contributed to PhysioNet in September 2004 by its creators Michael Oeff, Hans Koch, Ralf Bousseljot, and Dieter Kreiseler of the Physikalisch-Technische Bundesanstalt in Berlin [22,23].

The objective of the present article is to create, according to the CSE protocol, a set of manually measured QT intervals for the PTB Diagnostic ECG Database recordings. A fully accessible Internet reference set will provide a standard for the development of automated methods for QT interval measurement, as well as for Q-onsets and T-wave ends markings.

## Method

### Database

Each of the 549 recordings contains 15 simultaneously acquired signals: the conventional 12 leads and the 3 Frank (XYZ) leads. All leads are digitized at 1000 samples per second, with 16 bit resolution, over a range of ± 16.384 mV. The recordings come from 294 subjects (each represented by 1 to 5 recordings) with a broad range of ages and diagnoses. About 20 % of the subjects are healthy controls. The recordings are each typically about 2 min in length, with a small number of shorter recordings (none less than 30 s).

Each ECG recording is accompanied by a detailed clinical summary, including age, gender, diagnosis, and where applicable, data on medical history, medication and interventions, coronary artery pathology, ventriculography, echocardiography, and hemodynamics [21]. Diagnostic classification of the subjects include: myocardial infarction, cardiomyopathy/heart failure, bundle branch block, dysrhythmia, myocardial hypertrophy, etc., are also described.

### Study protocol

The PTB Diagnostic ECG Database was given to four cardiologists and one biomedical engineer (the experts) for manual marking of the QRS onset and the end of the T wave.

Each recording contained one selected beat, chosen visually by one of the referees, in such a way as to be close to the dominant beat (assumed as 'normal') with the least possible baseline shift, noise, and artifact.

**Figure 1:**
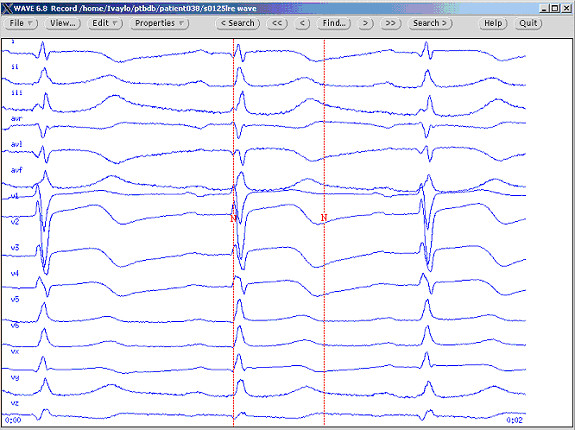
PTB Diagnostic ECG Database visualized by the Wave 6.8 program. Example of individual marking of QRS onset and T-wave end in lead II (the red vertical tracings).

The ECG recordings were visualized and marked by the help of the program Wave 6.8 freely accessible on the Internet [21]. This program illustrates all the 15 simultaneous recordings: the conventional 12 leads and the 3 Frank (XYZ) leads (Fig. 1). The selectable time scale was set to 125 mm/s and the amplitude scale to 20 mm/mV for all the experts.

Following the Challenge 2006 recommendations [21], the experts were instructed to mark the Q-onset and T-wave end in one of the leads only (lead II in the case). Such a measurement concept may also contribute to evaluation of the QT dispersion and is illustrated in Fig. 1 where the Q-onset mark in lead II (the red vertical line) is later than it would be if a group-QRS onset had to be considered. The same can be seen for the T-wave end also – the mark in lead II is earlier than it would be for the group-T-wave end.

**Figure 2:**
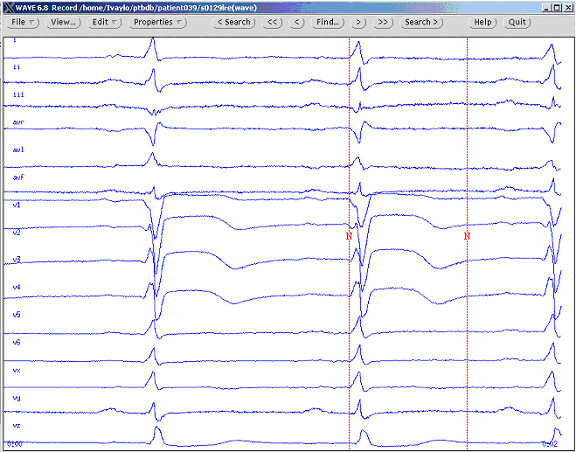
PTB Diagnostic ECG Database visualized by the Wave 6.8 program. Example of group marking of the T-wave end (the red vertical tracing) in cases when no T-wave in lead II can be observed or its amplitude is very small.

The semi-automated and fully-automated entries of the Challenge require measurements for at least 95 % (522) of the recordings. There is no instruction on what to do if no T wave could be definitely recognized in lead II. This was the case in more than 15 % of our recordings. In such a circumstance, the referees were instructed to mark the T-wave end as a group one at leads where the T wave is better manifested (see Fig. 2).

### Adjudication

Two deviation thresholds D1 and D2 were considered (Table 1), where D2, allowing more tolerance, was used solely in cases when only one of the referees differed considerably from the median [7].

**Table 1:** D1 and D2 thresholds in the measurements of the QRS onset and T-wave end

	QRS onset	T end
D1 ms	6	26
D2 ms	8	36

### Referees' analysis

As recommended by Willems *et al*. [7], the referee-cardiologists had experience in computer-assisted ECG interpretation, but to avoid bias, our referees had not been involved in program development. The biomedical engineer had never worked on precise Q-onset and T marking and was thus not influenced by his own methods.

**Figure 3:**
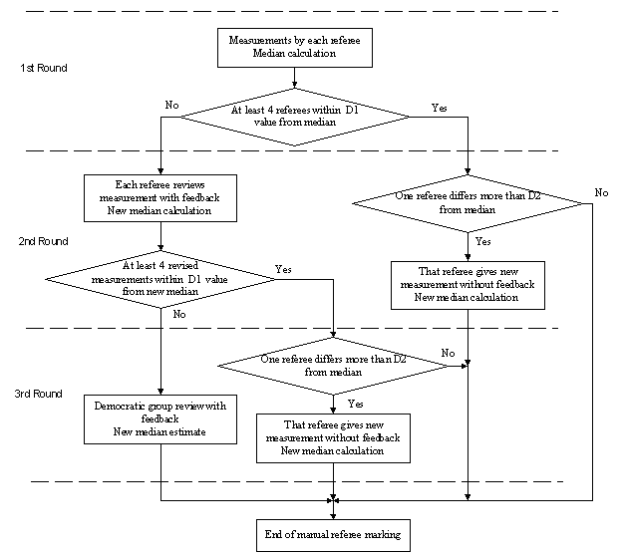
Reviewing rounds in the manual determination of the QRS onset and T-wave end.

A modified Delphi approach [24] and the study of Willems *et al*. [7] were used in the part concerning the manual measurement of characteristic ECG waves (Fig. 3). The three rounds include the initial and the subsequent two referees measurements (if necessary) to obtain results closer to the median.

**Figure 4:**
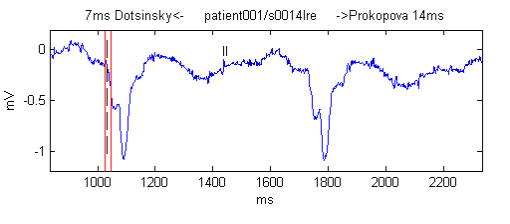
Example of the feedback forwarded to all the referees during 2nd and 3rd rounds. The black vertical dashed line is the median. The red vertical lines denote the most left and the most right marks made by the observers, with their name and deviation from the median shown as a text at the top of the figure.

Before proceeding to the 2nd and 3rd rounds, each referee received feedback for a limited amount of time. The referee was shown the median and the left-most and the right-most markings along with the respective person who had generated them (Fig. 4). Once shown and individually analyzed by each of the observers, all markings were hidden, and no further observations to the feedback allowed.

## Results

A program in Matlab was developed for calculation of the referees' median and to control the reviewing of the QRS onset and T-wave end markings during the 1st, 2nd and 3rd rounds (see block diagram of Fig. 3).

### Round 1

The Q-onset and T-wave end were marked in all recordings except for patient285/s0544_re, where no ECG-like tracings were observed. The total number of manual QT intervals marked was 2740 (548 recordings * 5 referees) read by the program Wave 6.8 [21]. The condition 'at least 4 referees within D1 value from median' (Fig. 3) was fulfilled for both Q and T marks for 495 recordings, while 40 cases of Q-onsets and 13 of T-wave ends did not meet the requirement.

### Round 2

All 5 referees repeated the 40+13 measurements using feedback of the corresponding median. The condition 'at least 4 revised measurements within D1 value from new median' was fulfilled for 33 Q-onsets and 7 T-wave ends. 7 Q-onsets and 6 T-wave ends did not meet the requirement.

The second condition, 'one referee differs more than D2 from the median' compelled this referee to give a new mark: 35 times for the Q-onset and 23 times for the T-wave end. A new median was then calculated.

The total number of the referees' corrections during the 2nd round was 40*5+35 = 235 for the Q-onset and 13*5+23 = 88 for the T-wave end, which represents, respectively, 8.58 % and 3.21 % of the total of 548 markings.

### Round 3

The 7 Q-onsets and 6 T-wave ends that failed consensus in Round 2 were reviewed by a 'Democratic' group process in a 3rd round. To reach a decision for the Democratic group, the outlying expert was shown the median of the 4 other experts. He was then free to mark anywhere, even to repeat his original outlying choice, and a new median was calculated.

One referee differed more than D2 from the median in 6 cases of Q-onset and 2 cases of T-wave end, and he repeated the markings with no feedback (this approach is discussed in the next section), following the protocol of the CSE [7]. The results were included in the statistics.

The total marking of Q-onsets repeated in the third round was 7*5+6 = 41 or 1.50 %. The corresponding results for the T-wave end was 6*5+2 = 32 or 1.17 %.

The maximal, mean, and standard deviations from the absolute differences between the median (assumed as the 'truth') and the referees' markings for each round are given in Table 2.  is the maximal possible error in the 99 % confidence interval calculated according to the Student's distribution. Here, *s *stands for the standard deviation and *n *is the total number of recordings.

**Figure 5:**
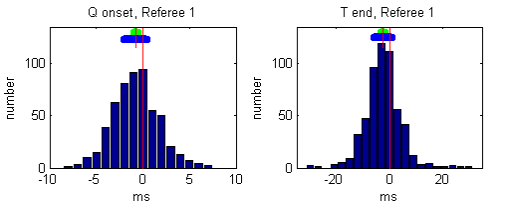
Histogram of deviations between the markings of Referee 1 and the Q-onset and T-wave end medians. The mean referees' deviation is depicted by small red vertical line, the 99 % confidence interval is presented by green horizontal bar and the standard deviation is given by blue horizontal bar.

**Figure 6:**
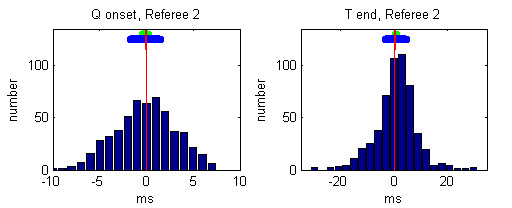
Histogram of deviations between the markings of Referee 2 and the Q-onset and T-wave end medians. The mean referees' deviation is depicted by small red vertical line, the 99 % confidence interval is presented by green horizontal bar and the standard deviation is given by blue horizontal bar.

**Figure 7:**
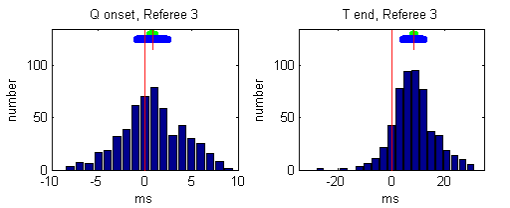
Histogram of deviations between the markings of Referee 3 and the Q-onset and T-wave end medians. The mean referees' deviation is depicted by short red vertical line, the 99 % confidence interval is presented by green horizontal bar and the standard deviation is given by blue horizontal bar.

**Figure 8:**
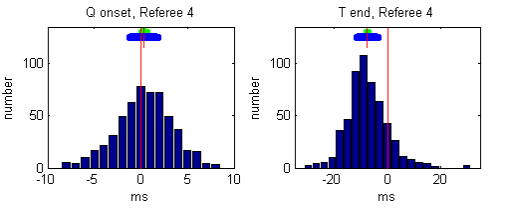
Histogram of deviations between the markings of Referee 4 and the Q-onset and T-wave end medians. The mean referees' deviation is depicted by small red vertical line, the 99 % confidence interval is presented by green horizontal bar and the standard deviation is given by blue horizontal bar.

**Figure 9:**
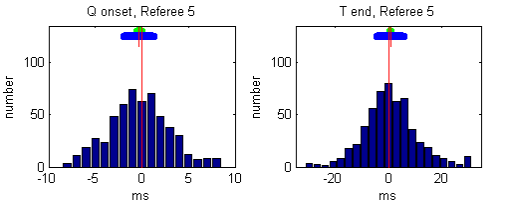
Histogram of deviations between the markings of Referee 5 and the Q-onset and T-wave end medians. The mean referees' deviation is depicted by small red vertical line, the 99 % confidence interval is presented by green horizontal bar and the standard deviation is given by blue horizontal bar.

Individual histograms of the referees' deviations from the median values after the 3rd round are presented in Figures 5, 6, 7, 8, 9.

**Table 2:** Maximal, mean and standard deviations from the absolute differences between the median and the referees' markings for each round.

	Q onset deviations [ms]				T end deviations [ms]			
	
	Max	Mean	Standard	Δ_0.01_	Max	Mean	Standard	Δ_0.01_
Round 1	24	2.83	1.51	0.28	75	8.21	5.23	0.96
Round 2	19	2.50	1.07	0.20	89	7.62	3.94	0.72
Round 3	11	2.43	0.96	0.18	39	7.43	3.44	0.63

The mean and the standard deviations of each observer after the 3rd round are given in Table 3:

**Table 3:** Mean and standard deviations of each referee after the 3rd round

	Q onset deviations [ms]		T end deviations [ms]	
	
	Mean	Standard	Mean	Standard
Referee 1	-0.76	2.45	-2.45	6.72
Referee 2	-0.11	3.23	0.75	7.62
Referee 3	0.81	3.29	8.43	7.88
Referee 4	0.34	2.99	-7.59	7.68
Referee 5	-0.29	3.22	0.78	10.24

Individual measurements, as well as the median estimation after the 3rd round for all recordings in the PTB Diagnostic ECG Database are presented in Table 4 (see additional file 1).

The data in the table is presented as time in milliseconds measured from the beginning of the record.

## Discussion

**Figure 10:**
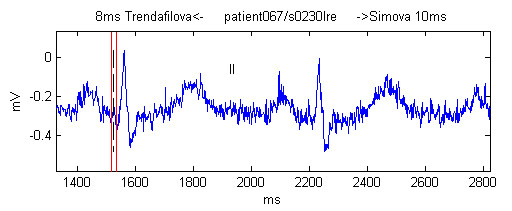
Example of manual marking in presence of electromyographic noise. The black vertical dashed line is the median. The red vertical lines denote the most left and right markings made by the observers, with their name and deviation from the median shown as a text at the top of the figure.

**Figure 11:**
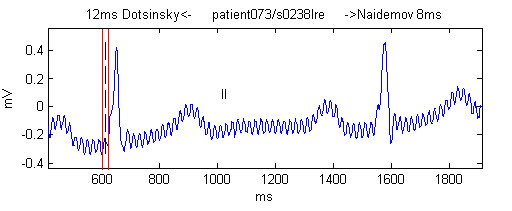
Example of referees marking in presence of mains interference. The black vertical dashed line is the median. The red vertical lines denote the most left and right markings made by the observers, with their name and deviation from the median shown as a text at the top of the figure.

Willems *et al*. [7] found that their referee's corrections of the Q-onset during the second round were 7.8 % of the total of Q-onset markings. This was slightly less than our result of 8.58 %, but is apparently not statistically different. Our result is mostly due to the fact that we used no signal preprocessing and some of our recordings were contaminated with electromyographic noise (Figure 10) or mains interference (Figure 11). For this reason it was extremely difficult to obtain a result closer to the median.

We made 3.21 % second round corrections for the T-wave end, which is almost three times lower compared to 9.0 % by Willems *et al*. [7]. The tolerances D1 = 26 ms and D2 = 36 ms [7] for the T-wave end are so large that a misfit caused by the mains interference having a period of 20 ms or by bursts of electromyographic noise of less than 15 ms did not require repetition in measurements. One can speculate that perhaps the small number of corrections during the 2nd and the 3rd rounds is due to the our protocol allowing for a group-marking of the T-wave end when no T wave in lead II can be observed or its amplitude is very small. Another reason may be certain differences between the databases: especially selected ECGs (which perhaps are more complicated for marking of the T-wave end) in Willems *et al*. [7], compared to the whole PTB Diagnostic ECG Database in our study.

The small number of corrections for the T-wave end in the 2nd and 3rd rounds, made us think that the tolerances used can be reduced, thus leading to lower dispersion of the manual T-wave end marking.

The idea to eliminate individual referee outliers differing considerably from the estimated median in successive steps was adopted from the work of Willems *et al*. [7]. However, there is something incomplete when, after the 3rd round, there is a referee differing more than D2 from the median. For this case, no more measurements are proposed and manual marking is completed with the inclusion of the 'wrong' or outlying measurement in the median estimation. Such an event appeared in patient023/s0085lre, patient097/s0384lre and patient208/s0430_re for the Q-onset and patient023/s0080lre for the T-wave end (Table 4, see additional file: 1). Our opinion is that the 'democratic decision' in cases where less than four referees' measurements are within D1 from the median can also be applied to the situation where one referee differs more than D2 from the median. The democratic decision is to exclude this 'wrong' referee from the median estimation.

## Conclusion

A fully accessible, on the Internet, dataset of manually measured Q-onsets and T-wave ends was created and presented in Table 4 of additional file 1 with this article. Thus, an available standard can be used for the development of automated methods for the detection of Q-onsets, T-wave ends and for QT interval measurements.

## Supplementary Material

Additional File 1 Table 4 is in additional file 1. Click here for file
