# Supplementary material for: Dataset of manually measured QT intervals in the electrocardiogram
Source: Biomed Eng Online. 2006 May 18;5:31. doi: 10.1186/1475-925X-5-31 (PMC1524770; doi:10.1186/1475-925X-5-31)
Supplement: Additional File 1 — Table 4 is in additional file 1. [file 1475-925X-5-31-S1.doc]

# Additional file 1

Table 4.

Dataset of individual referees’ measurements (R1, R2, R3, R4, R5) and the median (Med in blue color) of all recordings after the 3rd round in the PTB Diagnostic ECG Database, except for patient285/s0544_re, where no ECG-like tracings were observed. The data in the table is presented as time in milliseconds measured from the beginning of the record.

|  |  | Q onset | | | | | | T end | | | | | |
| --- | --- | --- | --- | --- | --- | --- | --- | --- | --- | --- | --- | --- | --- |
|  |  | R 1 | R 2 | R 3 | R 4 | R 5 | **Med** | R 1 | R 2 | R 3 | R 4 | R 5 | **Med** |
| patient1 | s0010_re | 1346 | 1342 | 1336 | 1340 | 1337 | **1340** | 1755 | 1756 | 1788 | 1742 | 1740 | **1756** |
|  | s0014lre | 1028 | 1031 | 1027 | 1032 | 1021 | **1028** | 1446 | 1450 | 1474 | 1441 | 1444 | **1451** |
|  | s0016lre | 1116 | 1120 | 1116 | 1117 | 1109 | **1116** | 1552 | 1555 | 1579 | 1554 | 1543 | **1557** |
| patient2 | s0015lre | 1384 | 1380 | 1384 | 1380 | 1380 | **1382** | 1728 | 1735 | 1764 | 1724 | 1728 | **1736** |
| patient3 | s0017lre | 854 | 860 | 859 | 855 | 851 | **856** | 1209 | 1205 | 1243 | 1220 | 1233 | **1222** |
| patient4 | s0020are | 732 | 741 | 728 | 734 | 727 | **732** | 1075 | 1066 | 1073 | 1059 | 1075 | **1070** |
|  | s0020bre | 949 | 962 | 956 | 951 | 949 | **953** | 1315 | 1320 | 1330 | 1318 | 1299 | **1316** |
| patient5 | s0021are | 1016 | 1025 | 1021 | 1017 | 1021 | **1020** | 1292 | 1290 | 1317 | 1285 | 1292 | **1295** |
|  | s0021bre | 763 | 763 | 758 | 752 | 752 | **758** | 1053 | 1066 | 1075 | 1077 | 1050 | **1064** |
|  | s0025lre | 596 | 598 | 598 | 601 | 592 | **597** | 881 | 883 | 883 | 874 | 885 | **881** |
|  | s0031lre | 833 | 849 | 840 | 845 | 838 | **841** | 1159 | 1154 | 1170 | 1157 | 1157 | **1159** |
|  | s0101lre | 1123 | 1129 | 1123 | 1120 | 1120 | **1123** | 1518 | 1500 | 1518 | 1520 | 1505 | **1512** |
| patient6 | s0022lre | 1195 | 1199 | 1196 | 1193 | 1204 | **1197** | 1525 | 1527 | 1550 | 1527 | 1529 | **1532** |
|  | s0027lre | 1369 | 1371 | 1360 | 1369 | 1367 | **1367** | 1737 | 1726 | 1739 | 1726 | 1753 | **1736** |
|  | s0064lre | 827 | 824 | 821 | 824 | 827 | **825** | 1186 | 1184 | 1189 | 1168 | 1168 | **1179** |
| patient7 | s0026lre | 799 | 803 | 803 | 800 | 803 | **802** | 1161 | 1152 | 1173 | 1152 | 1195 | **1167** |
|  | s0029lre | 804 | 811 | 807 | 807 | 806 | **807** | 1179 | 1159 | 1184 | 1163 | 1184 | **1174** |
|  | s0038lre | 1369 | 1371 | 1368 | 1369 | 1369 | **1369** | 1749 | 1737 | 1758 | 1728 | 1735 | **1741** |
|  | s0078lre | 1315 | 1322 | 1314 | 1319 | 1308 | **1316** | 1746 | 1735 | 1766 | 1717 | 1724 | **1738** |
| patient8 | s0028lre | 1179 | 1195 | 1182 | 1181 | 1181 | **1184** | 1593 | 1604 | 1615 | 1590 | 1588 | **1598** |
|  | s0037lre | 1489 | 1502 | 1493 | 1489 | 1490 | **1493** | 1914 | 1923 | 1948 | 1914 | 1902 | **1920** |
|  | s0068lre | 2048 | 2046 | 2046 | 2047 | 2041 | **2046** | 2466 | 2479 | 2471 | 2481 | 2466 | **2473** |
| patient9 | s0035_re | 958 | 960 | 958 | 962 | 953 | **958** | 1319 | 1318 | 1333 | 1310 | 1319 | **1320** |
| patient10 | s0036lre | 734 | 738 | 734 | 732 | 727 | **733** | 1098 | 1105 | 1116 | 1089 | 1089 | **1099** |
|  | s0042lre | 1041 | 1046 | 1040 | 1041 | 1041 | **1042** | 1439 | 1446 | 1441 | 1421 | 1421 | **1434** |
|  | s0061lre | 1231 | 1236 | 1230 | 1231 | 1229 | **1231** | 1595 | 1595 | 1612 | 1579 | 1577 | **1592** |
| patient11 | s0039lre | 935 | 935 | 934 | 931 | 928 | **933** | 1308 | 1312 | 1317 | 1306 | 1292 | **1307** |
|  | s0044lre | 958 | 960 | 959 | 958 | 953 | **958** | 1319 | 1326 | 1330 | 1308 | 1308 | **1318** |
|  | s0049lre | 743 | 748 | 739 | 742 | 743 | **743** | 1114 | 1110 | 1127 | 1084 | 1102 | **1107** |
|  | s0067lre | 1236 | 1238 | 1230 | 1234 | 1231 | **1234** | 1606 | 1615 | 1623 | 1602 | 1599 | **1609** |
| patient12 | s0043lre | 1466 | 1464 | 1457 | 1468 | 1462 | **1463** | 1884 | 1900 | 1888 | 1877 | 1898 | **1889** |
|  | s0050lre | 946 | 951 | 943 | 951 | 944 | **947** | 1303 | 1303 | 1317 | 1294 | 1308 | **1305** |
| patient13 | s0045lre | 1032 | 1035 | 1037 | 1030 | 1028 | **1032** | 1378 | 1385 | 1384 | 1362 | 1369 | **1376** |
|  | s0051lre | 1407 | 1405 | 1406 | 1405 | 1401 | **1405** | 1735 | 1740 | 1750 | 1724 | 1737 | **1737** |
|  | s0072lre | 1105 | 1100 | 1094 | 1102 | 1102 | **1101** | 1450 | 1455 | 1452 | 1437 | 1441 | **1447** |
| patient14 | s0046lre | 1310 | 1310 | 1310 | 1308 | 1301 | **1308** | 1665 | 1672 | 1672 | 1661 | 1674 | **1669** |
|  | s0056lre | 1579 | 1584 | 1577 | 1579 | 1579 | **1580** | 1981 | 1968 | 1986 | 1970 | 1963 | **1974** |
|  | s0071lre | 1166 | 1170 | 1165 | 1166 | 1163 | **1166** | 1525 | 1532 | 1531 | 1511 | 1529 | **1526** |
| patient15 | s0047lre | 1668 | 1666 | 1666 | 1668 | 1664 | **1666** | 2003 | 2010 | 2016 | 2005 | 2035 | **2014** |
|  | s0057lre | 1292 | 1294 | 1290 | 1294 | 1292 | **1292** | 1654 | 1667 | 1663 | 1649 | 1656 | **1658** |
|  | s0152lre | 1627 | 1629 | 1626 | 1629 | 1627 | **1628** | 1969 | 1988 | 1994 | 1963 | 1972 | **1977** |
| patient16 | s0052lre | 560 | 564 | 561 | 560 | 555 | **560** | 935 | 937 | 948 | 924 | 924 | **934** |
|  | s0060lre | 1019 | 1025 | 1016 | 1018 | 1019 | **1019** | 1394 | 1403 | 1425 | 1374 | 1378 | **1395** |
|  | s0076lre | 908 | 903 | 907 | 910 | 906 | **907** | 1285 | 1299 | 1300 | 1270 | 1279 | **1287** |
| patient17 | s0053lre | 1166 | 1170 | 1178 | 1172 | 1172 | **1172** | 1493 | 1502 | 1509 | 1493 | 1487 | **1497** |
|  | s0055lre | 1493 | 1498 | 1506 | 1493 | 1496 | **1497** | 1841 | 1842 | 1850 | 1825 | 1864 | **1844** |
|  | s0063lre | 1163 | 1161 | 1165 | 1163 | 1166 | **1164** | 1482 | 1498 | 1493 | 1480 | 1507 | **1492** |
|  | s0075lre | 1342 | 1346 | 1357 | 1349 | 1344 | **1348** | 1710 | 1715 | 1715 | 1706 | 1703 | **1710** |
| patient18 | s0054lre | 890 | 897 | 889 | 897 | 894 | **893** | 1254 | 1265 | 1257 | 1238 | 1254 | **1254** |
|  | s0059lre | 1236 | 1242 | 1238 | 1236 | 1240 | **1238** | 1627 | 1630 | 1628 | 1618 | 1618 | **1624** |
|  | s0082lre | 865 | 870 | 869 | 870 | 870 | **869** | 1263 | 1267 | 1279 | 1256 | 1258 | **1265** |
| patient19 | s0058lre | 1297 | 1299 | 1300 | 1301 | 1301 | **1300** | 1633 | 1640 | 1636 | 1624 | 1645 | **1636** |
|  | s0070lre | 1270 | 1274 | 1273 | 1272 | 1265 | **1271** | 1602 | 1566 | 1609 | 1599 | 1609 | **1597** |
|  | s0077lre | 1231 | 1233 | 1230 | 1236 | 1224 | **1231** | 1597 | 1622 | 1607 | 1597 | 1602 | **1605** |
| patient20 | s0062lre | 788 | 793 | 794 | 794 | 783 | **790** | 1211 | 1197 | 1222 | 1222 | 1213 | **1213** |
|  | s0069lre | 1262 | 1251 | 1260 | 1254 | 1255 | **1256** | 1685 | 1667 | 1704 | 1674 | 1670 | **1680** |
|  | s0079lre | 1444 | 1432 | 1442 | 1446 | 1440 | **1441** | 1785 | 1778 | 1783 | 1764 | 1776 | **1777** |
| patient21 | s0065lre | 1168 | 1172 | 1170 | 1170 | 1163 | **1169** | 1536 | 1554 | 1539 | 1539 | 1552 | **1544** |
|  | s0073lre | 1075 | 1080 | 1073 | 1073 | 1075 | **1075** | 1444 | 1432 | 1447 | 1441 | 1441 | **1441** |
|  | s0097lre | 1303 | 1308 | 1306 | 1306 | 1301 | **1305** | 1676 | 1667 | 1682 | 1682 | 1672 | **1676** |
| patient22 | s0066lre | 1319 | 1322 | 1322 | 1324 | 1315 | **1320** | 1692 | 1685 | 1707 | 1679 | 1683 | **1689** |
|  | s0074lre | 1331 | 1331 | 1331 | 1333 | 1324 | **1330** | 1703 | 1676 | 1718 | 1690 | 1692 | **1696** |
|  | s0149lre | 1098 | 1093 | 1096 | 1098 | 1087 | **1094** | 1464 | 1460 | 1476 | 1455 | 1462 | **1463** |
| patient23 | s0080lre | 1023 | 1025 | 1020 | 1025 | 1021 | **1023** | 1430 | 1385 | 1432 | 1416 | 1384 | **1409** |
|  | s0081lre | 989 | 985 | 991 | 983 | 985 | **987** | 1374 | 1358 | 1379 | 1355 | 1374 | **1368** |
|  | s0085lre | 1172 | 1168 | 1181 | 1166 | 1175 | **1172** | 1597 | 1586 | 1599 | 1581 | 1590 | **1591** |
|  | s0103lre | 1563 | 1564 | 1571 | 1563 | 1559 | **1564** | 1994 | 1989 | 2021 | 1979 | 1986 | **1994** |
| patient24 | s0083lre | 1514 | 1516 | 1523 | 1511 | 1509 | **1515** | 1887 | 1902 | 1899 | 1873 | 1887 | **1890** |
|  | s0084lre | 1202 | 1195 | 1201 | 1195 | 1200 | **1199** | 1541 | 1536 | 1558 | 1532 | 1545 | **1542** |
|  | s0086lre | 1663 | 1662 | 1663 | 1654 | 1650 | **1658** | 2019 | 2010 | 2043 | 2019 | 2021 | **2022** |
|  | s0094lre | 933 | 932 | 932 | 928 | 930 | **931** | 1272 | 1281 | 1303 | 1267 | 1283 | **1281** |
| patient25 | s0087lre | 2351 | 2350 | 2352 | 2345 | 2351 | **2350** | 2758 | 2758 | 2766 | 2758 | 2776 | **2763** |
|  | s0091lre | 1953 | 1960 | 1959 | 1959 | 1953 | **1957** | 2370 | 2378 | 2363 | 2363 | 2360 | **2367** |
|  | s0150lre | 1102 | 1102 | 1105 | 1102 | 1100 | **1102** | 1525 | 1518 | 1547 | 1507 | 1548 | **1529** |
| patient26 | s0088lre | 1091 | 1096 | 1094 | 1096 | 1087 | **1093** | 1487 | 1489 | 1495 | 1473 | 1484 | **1486** |
|  | s0095lre | 1560 | 1566 | 1563 | 1561 | 1559 | **1562** | 1969 | 1972 | 1978 | 1961 | 1990 | **1974** |
| patient27 | s0089lre | 770 | 781 | 780 | 784 | 775 | **778** | 1075 | 1120 | 1084 | 1089 | 1098 | **1093** |
|  | s0096lre | 802 | 800 | 810 | 813 | 802 | **805** | 1100 | 1089 | 1108 | 1089 | 1120 | **1101** |
|  | s0151lre | 912 | 922 | 924 | 912 | 922 | **918** | 1238 | 1261 | 1246 | 1227 | 1272 | **1249** |
| patient28 | s0090lre | 553 | 551 | 563 | 555 | 558 | **556** | 962 | 949 | 978 | 937 | 942 | **954** |
|  | s0093lre | 795 | 795 | 804 | 797 | 797 | **798** | 1206 | 1199 | 1222 | 1188 | 1209 | **1205** |
|  | s0108lre | 492 | 497 | 495 | 497 | 492 | **495** | 908 | 910 | 934 | 897 | 922 | **914** |
| patient29 | s0092lre | 788 | 790 | 789 | 788 | 781 | **787** | 1129 | 1145 | 1170 | 1125 | 1186 | **1151** |
|  | s0098lre | 3892 | 3890 | 3894 | 3898 | 3885 | **3892** | 4315 | 4306 | 4325 | 4303 | 4303 | **4310** |
|  | s0122lre | 978 | 980 | 981 | 976 | 976 | **978** | 1380 | 1374 | 1384 | 1340 | 1353 | **1366** |
| patient30 | s0099lre | 1010 | 1007 | 1021 | 1005 | 1007 | **1010** | 1396 | 1403 | 1414 | 1380 | 1392 | **1397** |
|  | s0107lre | 1326 | 1326 | 1333 | 1326 | 1322 | **1327** | 1749 | 1753 | 1756 | 1731 | 1758 | **1749** |
|  | s0117lre | 1294 | 1297 | 1306 | 1301 | 1294 | **1298** | 1778 | 1769 | 1726 | 1726 | 1760 | **1752** |
|  | s0153lre | 1190 | 1190 | 1192 | 1190 | 1193 | **1191** | 1590 | 1585 | 1595 | 1575 | 1577 | **1584** |
| patient31 | s0100lre | 1231 | 1238 | 1235 | 1238 | 1229 | **1234** | 1570 | 1586 | 1574 | 1557 | 1586 | **1575** |
|  | s0104lre | 811 | 813 | 811 | 815 | 809 | **812** | 1161 | 1163 | 1168 | 1166 | 1163 | **1164** |
|  | s0114lre | 858 | 865 | 859 | 861 | 856 | **860** | 1199 | 1209 | 1203 | 1190 | 1211 | **1202** |
|  | s0127lre | 1114 | 1117 | 1116 | 1116 | 1125 | **1118** | 1480 | 1487 | 1493 | 1464 | 1471 | **1479** |
| patient32 | s0102lre | 1059 | 1062 | 1054 | 1064 | 1066 | **1061** | 1383 | 1387 | 1403 | 1383 | 1374 | **1386** |
|  | s0106lre | 1303 | 1308 | 1306 | 1304 | 1308 | **1306** | 1620 | 1622 | 1642 | 1622 | 1667 | **1635** |
|  | s0115lre | 1125 | 1127 | 1124 | 1125 | 1134 | **1127** | 1532 | 1534 | 1542 | 1498 | 1534 | **1528** |
|  | s0165lre | 788 | 790 | 789 | 784 | 790 | **788** | 1161 | 1163 | 1165 | 1170 | 1157 | **1163** |
| patient33 | s0105lre | 1319 | 1320 | 1319 | 1322 | 1317 | **1319** | 1651 | 1650 | 1672 | 1647 | 1697 | **1663** |
|  | s0113lre | 741 | 745 | 743 | 745 | 743 | **743** | 1127 | 1132 | 1129 | 1107 | 1114 | **1122** |
|  | s0121lre | 1233 | 1238 | 1233 | 1231 | 1229 | **1233** | 1615 | 1612 | 1634 | 1597 | 1606 | **1613** |
|  | s0157lre | 969 | 976 | 970 | 969 | 971 | **971** | 1358 | 1360 | 1360 | 1355 | 1360 | **1359** |
| patient34 | s0109lre | 881 | 883 | 882 | 872 | 881 | **880** | 1172 | 1163 | 1172 | 1175 | 1177 | **1172** |
|  | s0118lre | 1249 | 1248 | 1249 | 1249 | 1251 | **1249** | 1559 | 1561 | 1571 | 1568 | 1577 | **1567** |
|  | s0123lre | 1401 | 1403 | 1401 | 1398 | 1401 | **1401** | 1744 | 1746 | 1750 | 1737 | 1751 | **1746** |
|  | s0158lre | 1301 | 1303 | 1300 | 1301 | 1299 | **1301** | 1645 | 1651 | 1655 | 1636 | 1649 | **1647** |
| patient35 | s0110lre | 1032 | 1041 | 1027 | 1030 | 1044 | **1035** | 1385 | 1392 | 1401 | 1380 | 1401 | **1392** |
|  | s0119lre | 1245 | 1248 | 1241 | 1247 | 1256 | **1247** | 1618 | 1609 | 1615 | 1599 | 1624 | **1613** |
|  | s0124lre | 881 | 888 | 875 | 879 | 883 | **881** | 1224 | 1218 | 1227 | 1213 | 1240 | **1224** |
|  | s0145lre | 684 | 687 | 677 | 687 | 680 | **683** | 1055 | 1059 | 1078 | 1053 | 1064 | **1062** |
| patient36 | s0111lre | 1401 | 1396 | 1400 | 1394 | 1389 | **1396** | 1789 | 1792 | 1791 | 1776 | 1785 | **1787** |
|  | s0116lre | 2731 | 2725 | 2723 | 2727 | 2729 | **2727** | 3102 | 3111 | 3113 | 3100 | 3116 | **3108** |
|  | s0126lre | 1229 | 1233 | 1222 | 1229 | 1224 | **1227** | 1627 | 1622 | 1647 | 1615 | 1624 | **1627** |
| patient37 | s0112lre | 1053 | 1053 | 1050 | 1053 | 1046 | **1051** | 1425 | 1428 | 1436 | 1421 | 1428 | **1428** |
|  | s0120lre | 1385 | 1380 | 1382 | 1385 | 1380 | **1382** | 1771 | 1762 | 1775 | 1753 | 1771 | **1766** |
| patient38 | s0125lre | 883 | 882 | 872 | 883 | 879 | **880** | 1227 | 1242 | 1243 | 1218 | 1236 | **1233** |
|  | s0128lre | 1344 | 1342 | 1333 | 1340 | 1340 | **1340** | 1699 | 1708 | 1704 | 1688 | 1692 | **1698** |
|  | s0162lre | 94 | 97 | 94 | 97 | 101 | **97** | 483 | 476 | 493 | 490 | 488 | **486** |
| patient39 | s0129lre | 1238 | 1240 | 1230 | 1238 | 1229 | **1235** | 1663 | 1657 | 1664 | 1666 | 1674 | **1665** |
|  | s0134lre | 1076 | 1080 | 1084 | 1077 | 1080 | **1079** | 1493 | 1498 | 1498 | 1489 | 1505 | **1497** |
|  | s0164lre | 793 | 793 | 799 | 793 | 793 | **794** | 1154 | 1161 | 1168 | 1157 | 1186 | **1165** |
| patient40 | s0130lre | 1190 | 1188 | 1191 | 1186 | 1181 | **1187** | 1502 | 1496 | 1517 | 1493 | 1543 | **1510** |
|  | s0131lre | 689 | 689 | 685 | 680 | 684 | **685** | 1050 | 1053 | 1059 | 1023 | 1046 | **1046** |
|  | s0133lre | 1362 | 1360 | 1357 | 1360 | 1360 | **1360** | 1737 | 1726 | 1739 | 1717 | 1737 | **1731** |
|  | s0219lre | 1554 | 1548 | 1552 | 1554 | 1548 | **1551** | 1936 | 1938 | 1937 | 1937 | 1941 | **1938** |
| patient41 | s0132lre | 974 | 971 | 970 | 976 | 974 | **973** | 1312 | 1306 | 1306 | 1283 | 1306 | **1303** |
|  | s0136lre | 1077 | 1080 | 1075 | 1080 | 1080 | **1078** | 1435 | 1431 | 1433 | 1425 | 1430 | **1431** |
|  | s0138lre | 806 | 809 | 807 | 811 | 811 | **809** | 1170 | 1175 | 1189 | 1159 | 1175 | **1174** |
|  | s0276lre | 820 | 831 | 823 | 827 | 824 | **825** | 1188 | 1197 | 1205 | 1175 | 1199 | **1193** |
| patient42 | s0135lre | 759 | 754 | 758 | 761 | 757 | **758** | 1087 | 1077 | 1094 | 1066 | 1080 | **1081** |
|  | s0137lre | 1213 | 1214 | 1205 | 1218 | 1211 | **1212** | 1559 | 1552 | 1560 | 1543 | 1552 | **1553** |
|  | s0140lre | 1166 | 1170 | 1168 | 1166 | 1163 | **1167** | 1543 | 1538 | 1547 | 1520 | 1532 | **1536** |
|  | s0347lre | 1254 | 1256 | 1260 | 1261 | 1256 | **1257** | 1647 | 1649 | 1655 | 1631 | 1649 | **1646** |
| patient43 | s0141lre | 759 | 759 | 758 | 763 | 754 | **759** | 1148 | 1138 | 1154 | 1127 | 1136 | **1141** |
|  | s0144lre | 1466 | 1463 | 1466 | 1464 | 1464 | **1465** | 1882 | 1866 | 1888 | 1857 | 1864 | **1871** |
|  | s0278lre | 1453 | 1457 | 1454 | 1454 | 1454 | **1454** | 1839 | 1844 | 1848 | 1825 | 1835 | **1838** |
| patient44 | s0142lre | 879 | 880 | 875 | 872 | 879 | **877** | 1247 | 1251 | 1257 | 1247 | 1251 | **1251** |
|  | s0143lre | 1091 | 1093 | 1084 | 1084 | 1091 | **1089** | 1473 | 1470 | 1476 | 1455 | 1484 | **1472** |
|  | s0146lre | 718 | 719 | 712 | 711 | 711 | **714** | 1132 | 1138 | 1146 | 1136 | 1141 | **1139** |
|  | s0159lre | 1681 | 1677 | 1682 | 1676 | 1673 | **1678** | 2114 | 2102 | 2138 | 2102 | 2120 | **2115** |
| patient45 | s0147lre | 1393 | 1398 | 1384 | 1396 | 1389 | **1392** | 1714 | 1724 | 1731 | 1708 | 1719 | **1719** |
|  | s0148lre | 698 | 702 | 696 | 705 | 700 | **700** | 1073 | 1055 | 1086 | 1068 | 1057 | **1068** |
|  | s0155lre | 917 | 919 | 915 | 922 | 919 | **918** | 1387 | 1374 | 1409 | 1380 | 1371 | **1384** |
|  | s0217lre | 876 | 883 | 872 | 879 | 874 | **877** | 1218 | 1193 | 1224 | 1181 | 1222 | **1208** |
| patient46 | s0156lre | 1005 | 1006 | 1013 | 1007 | 1014 | **1009** | 1376 | 1385 | 1365 | 1374 | 1389 | **1378** |
|  | s0161lre | 1206 | 1204 | 1211 | 1207 | 1206 | **1207** | 1529 | 1532 | 1544 | 1575 | 1541 | **1544** |
|  | s0168lre | 953 | 964 | 956 | 951 | 960 | **957** | 1279 | 1276 | 1287 | 1279 | 1276 | **1279** |
|  | s0184lre | 153 | 162 | 159 | 151 | 155 | **156** | 503 | 512 | 512 | 499 | 524 | **510** |
| patient47 | s0160lre | 1261 | 1276 | 1265 | 1270 | 1265 | **1267** | 1604 | 1618 | 1620 | 1590 | 1586 | **1604** |
|  | s0163lre | 1055 | 1056 | 1054 | 1055 | 1048 | **1054** | 1394 | 1412 | 1411 | 1385 | 1389 | **1398** |
|  | s0167lre | 1071 | 1066 | 1070 | 1071 | 1059 | **1067** | 1432 | 1462 | 1452 | 1462 | 1439 | **1449** |
| patient48 | s0171lre | 831 | 831 | 829 | 836 | 831 | **832** | 1188 | 1190 | 1195 | 1177 | 1195 | **1189** |
|  | s0172lre | 1385 | 1389 | 1387 | 1385 | 1385 | **1386** | 1769 | 1753 | 1780 | 1758 | 1776 | **1767** |
|  | s0180lre | 824 | 831 | 826 | 831 | 827 | **828** | 1301 | 1294 | 1311 | 1283 | 1290 | **1296** |
|  | s0277lre | 1322 | 1324 | 1322 | 1326 | 1326 | **1324** | 1722 | 1706 | 1734 | 1706 | 1717 | **1717** |
| patient49 | s0173lre | 1118 | 1116 | 1116 | 1118 | 1114 | **1116** | 1464 | 1460 | 1476 | 1453 | 1468 | **1464** |
|  | s0178lre | 987 | 980 | 981 | 978 | 983 | **982** | 1346 | 1358 | 1355 | 1337 | 1344 | **1348** |
|  | s0186lre | 1096 | 1089 | 1086 | 1089 | 1087 | **1089** | 1527 | 1529 | 1528 | 1507 | 1523 | **1523** |
|  | s0314lre | 1475 | 1466 | 1471 | 1468 | 1471 | **1470** | 1841 | 1842 | 1850 | 1825 | 1835 | **1839** |
| patient50 | s0174lre | 1199 | 1202 | 1192 | 1202 | 1202 | **1199** | 1584 | 1590 | 1596 | 1561 | 1581 | **1582** |
|  | s0177lre | 1581 | 1584 | 1579 | 1581 | 1584 | **1582** | 1960 | 1970 | 1970 | 1957 | 1977 | **1967** |
|  | s0185lre | 953 | 953 | 951 | 955 | 951 | **953** | 1342 | 1346 | 1349 | 1335 | 1349 | **1344** |
|  | s0215lre | 1511 | 1513 | 1511 | 1511 | 1509 | **1511** | 1914 | 1903 | 1912 | 1907 | 1923 | **1912** |
| patient51 | s0179lre | 1199 | 1197 | 1197 | 1199 | 1195 | **1197** | 1525 | 1548 | 1542 | 1518 | 1545 | **1536** |
|  | s0181lre | 922 | 919 | 922 | 910 | 919 | **918** | 1335 | 1324 | 1363 | 1326 | 1328 | **1335** |
|  | s0187lre | 951 | 950 | 953 | 944 | 946 | **949** | 1364 | 1371 | 1376 | 1369 | 1369 | **1370** |
|  | s0213lre | 1215 | 1211 | 1211 | 1215 | 1211 | **1213** | 1611 | 1597 | 1623 | 1618 | 1606 | **1611** |
| patient52 | s0190lre | 388 | 390 | 392 | 386 | 393 | **390** | 718 | 727 | 742 | 716 | 718 | **724** |
| patient53 | s0191lre | 1197 | 1197 | 1195 | 1199 | 1197 | **1197** | 1577 | 1588 | 1609 | 1566 | 1579 | **1584** |
| patient54 | s0192lre | 732 | 723 | 734 | 736 | 734 | **732** | 1093 | 1123 | 1105 | 1096 | 1089 | **1101** |
|  | s0195lre | 635 | 639 | 636 | 641 | 630 | **636** | 989 | 1003 | 1010 | 996 | 1003 | **1000** |
|  | s0197lre | 775 | 779 | 766 | 777 | 775 | **774** | 1143 | 1154 | 1162 | 1136 | 1132 | **1145** |
|  | s0218lre | 1254 | 1256 | 1257 | 1256 | 1251 | **1255** | 1656 | 1658 | 1650 | 1651 | 1649 | **1653** |
| patient55 | s0194lre | 781 | 779 | 777 | 784 | 781 | **780** | 1107 | 1111 | 1107 | 1109 | 1111 | **1109** |
| patient56 | s0196lre | 1093 | 1093 | 1094 | 1093 | 1096 | **1094** | 1529 | 1536 | 1550 | 1518 | 1527 | **1532** |
| patient57 | s0198lre | 1279 | 1277 | 1273 | 1280 | 1276 | **1277** | 1606 | 1601 | 1634 | 1602 | 1604 | **1609** |
| patient58 | s0216lre | 1064 | 1066 | 1065 | 1066 | 1065 | **1065** | 1453 | 1459 | 1468 | 1457 | 1439 | **1455** |
| patient59 | s0208lre | 1344 | 1349 | 1346 | 1351 | 1346 | **1347** | 1676 | 1717 | 1691 | 1663 | 1690 | **1687** |
| patient60 | s0209lre | 983 | 982 | 978 | 985 | 983 | **982** | 1324 | 1328 | 1325 | 1315 | 1333 | **1325** |
| patient61 | s0210lre | 833 | 832 | 837 | 829 | 833 | **833** | 1154 | 1161 | 1162 | 1157 | 1166 | **1160** |
| patient62 | s0212lre | 684 | 682 | 685 | 682 | 687 | **684** | 1091 | 1092 | 1100 | 1087 | 1091 | **1092** |
| patient63 | s0214lre | 1048 | 1043 | 1051 | 1055 | 1048 | **1049** | 1407 | 1414 | 1401 | 1387 | 1383 | **1398** |
| patient64 | s0220lre | 822 | 824 | 826 | 829 | 829 | **826** | 1168 | 1175 | 1181 | 1166 | 1172 | **1172** |
| patient65 | s0221lre | 865 | 870 | 872 | 870 | 867 | **869** | 1270 | 1283 | 1284 | 1270 | 1263 | **1274** |
|  | s0226lre | 734 | 734 | 737 | 736 | 732 | **735** | 1116 | 1125 | 1124 | 1116 | 1127 | **1122** |
|  | s0229lre | 1170 | 1171 | 1170 | 1172 | 1166 | **1170** | 1572 | 1579 | 1579 | 1575 | 1575 | **1576** |
|  | s0282lre | 1464 | 1465 | 1463 | 1462 | 1462 | **1463** | 1902 | 1911 | 1905 | 1893 | 1900 | **1902** |
| patient66 | s0225lre | 1231 | 1229 | 1230 | 1236 | 1231 | **1231** | 1585 | 1609 | 1617 | 1577 | 1604 | **1598** |
|  | s0231lre | 698 | 700 | 699 | 702 | 702 | **700** | 1071 | 1100 | 1111 | 1087 | 1091 | **1092** |
|  | s0280lre | 470 | 469 | 471 | 472 | 470 | **470** | 874 | 879 | 880 | 874 | 872 | **876** |
| patient67 | s0227lre | 436 | 433 | 444 | 440 | 440 | **439** | 677 | 693 | 696 | 673 | 689 | **686** |
|  | s0230lre | 1525 | 1523 | 1525 | 1523 | 1523 | **1524** | 1866 | 1884 | 1883 | 1862 | 1871 | **1873** |
|  | s0283lre | 718 | 720 | 718 | 718 | 720 | **719** | 1111 | 1112 | 1119 | 1109 | 1118 | **1114** |
| patient68 | s0228lre | 488 | 492 | 493 | 490 | 490 | **491** | 827 | 822 | 845 | 820 | 813 | **825** |
| patient69 | s0232lre | 1301 | 1296 | 1298 | 1301 | 1301 | **1299** | 1688 | 1697 | 1710 | 1685 | 1701 | **1696** |
|  | s0233lre | 761 | 761 | 764 | 761 | 766 | **763** | 1143 | 1152 | 1155 | 1148 | 1145 | **1149** |
|  | s0234lre | 894 | 890 | 895 | 895 | 899 | **895** | 1288 | 1297 | 1327 | 1310 | 1285 | **1301** |
|  | s0284lre | 738 | 738 | 734 | 736 | 741 | **737** | 1141 | 1150 | 1162 | 1145 | 1145 | **1149** |
| patient70 | s0235lre | 655 | 659 | 652 | 659 | 659 | **657** | 1007 | 1005 | 1013 | 1010 | 1005 | **1008** |
| patient71 | s0236lre | 368 | 370 | 368 | 368 | 372 | **369** | 797 | 813 | 829 | 806 | 813 | **812** |
| patient72 | s0237lre | 393 | 390 | 398 | 395 | 397 | **395** | 752 | 756 | 756 | 734 | 745 | **749** |
|  | s0240lre | 1028 | 1035 | 1024 | 1023 | 1032 | **1028** | 1448 | 1441 | 1455 | 1432 | 1446 | **1444** |
|  | s0244lre | 1206 | 1207 | 1203 | 1209 | 1206 | **1206** | 1627 | 1635 | 1639 | 1615 | 1638 | **1631** |
|  | s0318lre | 1093 | 1102 | 1094 | 1093 | 1100 | **1096** | 1493 | 1496 | 1504 | 1487 | 1491 | **1494** |
| patient73 | s0238lre | 610 | 614 | 601 | 612 | 616 | **611** | 983 | 994 | 1005 | 978 | 987 | **989** |
|  | s0243lre | 867 | 862 | 859 | 870 | 870 | **866** | 1236 | 1240 | 1249 | 1229 | 1238 | **1238** |
|  | s0249lre | 935 | 931 | 929 | 931 | 926 | **930** | 1301 | 1310 | 1317 | 1294 | 1331 | **1311** |
|  | s0252lre | 716 | 718 | 718 | 718 | 716 | **717** | 1084 | 1080 | 1100 | 1073 | 1091 | **1086** |
| patient74 | s0239lre | 653 | 653 | 652 | 655 | 650 | **653** | 998 | 1001 | 1013 | 996 | 1010 | **1004** |
|  | s0241lre | 958 | 955 | 956 | 960 | 953 | **956** | 1247 | 1251 | 1254 | 1236 | 1247 | **1247** |
|  | s0245lre | 558 | 554 | 552 | 562 | 560 | **557** | 849 | 865 | 859 | 840 | 892 | **861** |
|  | s0406lre | 996 | 989 | 991 | 998 | 994 | **994** | 1324 | 1328 | 1333 | 1317 | 1333 | **1327** |
| patient75 | s0242lre | 856 | 856 | 853 | 851 | 851 | **853** | 1247 | 1251 | 1254 | 1254 | 1274 | **1256** |
|  | s0246lre | 623 | 621 | 620 | 621 | 625 | **622** | 978 | 983 | 989 | 964 | 987 | **980** |
|  | s0248lre | 327 | 325 | 325 | 327 | 332 | **327** | 693 | 659 | 699 | 689 | 700 | **688** |
|  | s0327lre | 732 | 727 | 731 | 734 | 736 | **732** | 1073 | 1077 | 1089 | 1075 | 1080 | **1079** |
| patient76 | s0247lre | 1032 | 1035 | 1031 | 1035 | 1028 | **1032** | 1385 | 1394 | 1388 | 1369 | 1392 | **1386** |
|  | s0250lre | 833 | 827 | 831 | 836 | 833 | **832** | 1152 | 1145 | 1162 | 1150 | 1181 | **1158** |
|  | s0253lre | 668 | 664 | 669 | 668 | 666 | **667** | 1014 | 1014 | 1018 | 1012 | 1019 | **1015** |
|  | s0319lre | 917 | 919 | 918 | 919 | 915 | **918** | 1249 | 1254 | 1254 | 1249 | 1242 | **1250** |
| patient77 | s0251lre | 822 | 824 | 826 | 827 | 822 | **824** | 1143 | 1163 | 1146 | 1132 | 1172 | **1151** |
|  | s0254lre | 576 | 580 | 577 | 578 | 583 | **579** | 942 | 949 | 951 | 935 | 944 | **944** |
|  | s0258lre | 635 | 641 | 634 | 635 | 635 | **636** | 1014 | 1025 | 1027 | 1010 | 1039 | **1023** |
|  | s0285lre | 533 | 535 | 536 | 535 | 533 | **534** | 858 | 863 | 867 | 849 | 861 | **860** |
| patient78 | s0255lre | 1387 | 1387 | 1390 | 1392 | 1389 | **1389** | 1805 | 1807 | 1815 | 1792 | 1837 | **1811** |
|  | s0259lre | 1251 | 1258 | 1254 | 1258 | 1258 | **1256** | 1604 | 1615 | 1609 | 1593 | 1618 | **1608** |
|  | s0262lre | 955 | 964 | 955 | 960 | 967 | **960** | 1308 | 1315 | 1313 | 1294 | 1335 | **1313** |
|  | s0317lre | 1924 | 1924 | 1910 | 1922 | 1922 | **1920** | 2222 | 2238 | 2241 | 2218 | 2238 | **2231** |
| patient79 | s0256lre | 1032 | 1031 | 1024 | 1032 | 1028 | **1029** | 1416 | 1418 | 1428 | 1396 | 1410 | **1414** |
|  | s0257lre | 605 | 610 | 601 | 610 | 603 | **606** | 962 | 964 | 997 | 951 | 969 | **969** |
|  | s0263lre | 1340 | 1340 | 1341 | 1337 | 1337 | **1339** | 1728 | 1724 | 1728 | 1708 | 1742 | **1726** |
|  | s0269lre | 603 | 602 | 607 | 601 | 605 | **604** | 987 | 992 | 994 | 980 | 987 | **988** |
| patient80 | s0260lre | 1186 | 1188 | 1186 | 1188 | 1188 | **1187** | 1541 | 1505 | 1498 | 1496 | 1496 | **1507** |
|  | s0261lre | 763 | 763 | 766 | 768 | 766 | **765** | 1082 | 1046 | 1089 | 1039 | 1046 | **1060** |
|  | s0265lre | 1161 | 1160 | 1165 | 1159 | 1166 | **1162** | 1559 | 1551 | 1571 | 1550 | 1561 | **1558** |
|  | s0315lre | 650 | 655 | 653 | 655 | 655 | **654** | 994 | 1003 | 1013 | 994 | 1007 | **1002** |
| patient81 | s0264lre | 1450 | 1455 | 1457 | 1453 | 1457 | **1454** | 1810 | 1803 | 1818 | 1798 | 1825 | **1811** |
|  | s0266lre | 892 | 894 | 897 | 894 | 892 | **894** | 1297 | 1301 | 1306 | 1306 | 1306 | **1303** |
|  | s0270lre | 1457 | 1464 | 1455 | 1462 | 1464 | **1460** | 1830 | 1816 | 1840 | 1830 | 1832 | **1830** |
|  | s0346lre | 1114 | 1120 | 1119 | 1116 | 1125 | **1119** | 1487 | 1489 | 1491 | 1489 | 1487 | **1489** |
| patient82 | s0267lre | 718 | 720 | 715 | 723 | 725 | **720** | 1123 | 1125 | 1135 | 1123 | 1125 | **1126** |
|  | s0271lre | 1346 | 1342 | 1341 | 1344 | 1351 | **1345** | 1710 | 1715 | 1720 | 1710 | 1724 | **1716** |
|  | s0279lre | 1274 | 1270 | 1271 | 1267 | 1272 | **1271** | 1722 | 1706 | 1723 | 1719 | 1726 | **1719** |
|  | s0320lre | 1005 | 1007 | 1005 | 1010 | 1007 | **1007** | 1484 | 1448 | 1485 | 1462 | 1487 | **1473** |
| patient83 | s0268lre | 1396 | 1398 | 1401 | 1396 | 1403 | **1399** | 1776 | 1751 | 1758 | 1764 | 1760 | **1762** |
|  | s0272lre | 901 | 900 | 899 | 903 | 901 | **901** | 1186 | 1193 | 1197 | 1186 | 1193 | **1191** |
|  | s0286lre | 1096 | 1098 | 1094 | 1098 | 1098 | **1097** | 1473 | 1464 | 1479 | 1459 | 1462 | **1467** |
|  | s0290lre | 1380 | 1385 | 1382 | 1376 | 1380 | **1381** | 1753 | 1758 | 1764 | 1742 | 1742 | **1752** |
| patient84 | s0281lre | 1134 | 1136 | 1138 | 1125 | 1129 | **1132** | 1520 | 1534 | 1542 | 1514 | 1541 | **1530** |
|  | s0288lre | 736 | 738 | 739 | 727 | 736 | **735** | 1116 | 1125 | 1135 | 1116 | 1127 | **1124** |
|  | s0289lre | 863 | 865 | 864 | 861 | 863 | **863** | 1276 | 1267 | 1279 | 1265 | 1263 | **1270** |
|  | s0313lre | 1188 | 1190 | 1189 | 1188 | 1186 | **1188** | 1638 | 1642 | 1650 | 1627 | 1633 | **1638** |
| patient85 | s0296lre | 1276 | 1279 | 1275 | 1274 | 1285 | **1278** | 1658 | 1640 | 1674 | 1645 | 1672 | **1658** |
|  | s0297lre | 1213 | 1220 | 1214 | 1218 | 1213 | **1216** | 1658 | 1663 | 1680 | 1654 | 1667 | **1664** |
|  | s0298lre | 928 | 931 | 934 | 940 | 937 | **934** | 1376 | 1367 | 1392 | 1360 | 1378 | **1375** |
|  | s0345lre | 1538 | 1541 | 1542 | 1543 | 1545 | **1542** | 1936 | 1929 | 1945 | 1932 | 1929 | **1934** |
| patient86 | s0316lre | 1389 | 1392 | 1384 | 1389 | 1396 | **1390** | 1794 | 1783 | 1769 | 1769 | 1819 | **1787** |
| patient87 | s0321lre | 899 | 903 | 902 | 897 | 910 | **902** | 1342 | 1351 | 1349 | 1328 | 1331 | **1340** |
|  | s0326lre | 700 | 702 | 701 | 698 | 698 | **700** | 1048 | 1041 | 1059 | 1039 | 1044 | **1046** |
|  | s0330lre | 1188 | 1188 | 1192 | 1188 | 1186 | **1188** | 1500 | 1514 | 1504 | 1489 | 1514 | **1504** |
| patient88 | s0339lre | 1163 | 1166 | 1165 | 1166 | 1163 | **1165** | 1523 | 1527 | 1533 | 1518 | 1545 | **1529** |
|  | s0343lre | 431 | 436 | 430 | 433 | 442 | **434** | 788 | 779 | 796 | 775 | 784 | **784** |
|  | s0352lre | 809 | 811 | 810 | 811 | 813 | **811** | 1154 | 1161 | 1162 | 1145 | 1152 | **1155** |
|  | s0413lre | 951 | 953 | 951 | 953 | 951 | **952** | 1265 | 1270 | 1273 | 1258 | 1263 | **1266** |
| patient89 | s0344lre | 1012 | 1014 | 1012 | 1014 | 1005 | **1011** | 1398 | 1405 | 1405 | 1398 | 1410 | **1403** |
|  | s0355lre | 2299 | 2297 | 2300 | 2297 | 2299 | **2298** | 2654 | 2639 | 2669 | 2640 | 2645 | **2649** |
|  | s0359lre | 1581 | 1577 | 1585 | 1584 | 1575 | **1580** | 1957 | 1950 | 1964 | 1952 | 1972 | **1959** |
|  | s0372lre | 1172 | 1168 | 1170 | 1172 | 1166 | **1170** | 1541 | 1543 | 1552 | 1532 | 1543 | **1542** |
| patient90 | s0348lre | 881 | 888 | 883 | 881 | 881 | **883** | 1267 | 1274 | 1276 | 1251 | 1270 | **1268** |
|  | s0356lre | 892 | 899 | 899 | 897 | 901 | **898** | 1301 | 1292 | 1308 | 1297 | 1294 | **1298** |
|  | s0360lre | 845 | 849 | 848 | 845 | 851 | **848** | 1274 | 1276 | 1289 | 1265 | 1279 | **1277** |
|  | s0418lre | 687 | 689 | 688 | 680 | 682 | **685** | 1089 | 1093 | 1097 | 1077 | 1087 | **1089** |
| patient91 | s0353lre | 1154 | 1159 | 1157 | 1150 | 1161 | **1156** | 1584 | 1568 | 1569 | 1563 | 1552 | **1567** |
|  | s0357lre | 829 | 827 | 826 | 829 | 827 | **828** | 1179 | 1190 | 1186 | 1177 | 1190 | **1184** |
|  | s0361lre | 1123 | 1125 | 1119 | 1125 | 1123 | **1123** | 1484 | 1475 | 1485 | 1480 | 1471 | **1479** |
|  | s0408lre | 818 | 822 | 826 | 820 | 827 | **823** | 1236 | 1240 | 1246 | 1235 | 1218 | **1235** |
| patient92 | s0354lre | 1222 | 1227 | 1224 | 1227 | 1224 | **1225** | 1579 | 1575 | 1590 | 1572 | 1568 | **1577** |
|  | s0358lre | 1107 | 1107 | 1105 | 1107 | 1105 | **1106** | 1507 | 1509 | 1514 | 1509 | 1491 | **1506** |
|  | s0362lre | 677 | 675 | 680 | 675 | 675 | **676** | 1048 | 1057 | 1056 | 1057 | 1059 | **1055** |
|  | s0411lre | 976 | 983 | 983 | 983 | 976 | **980** | 1389 | 1387 | 1395 | 1387 | 1376 | **1387** |
| patient93 | s0367lre | 1163 | 1163 | 1168 | 1164 | 1161 | **1164** | 1511 | 1507 | 1520 | 1507 | 1505 | **1510** |
|  | s0371lre | 723 | 725 | 722 | 725 | 718 | **723** | 1059 | 1053 | 1073 | 1064 | 1046 | **1059** |
|  | s0375lre | 937 | 944 | 940 | 942 | 937 | **940** | 1294 | 1299 | 1306 | 1288 | 1290 | **1295** |
|  | s0378lre | 1258 | 1261 | 1254 | 1254 | 1256 | **1257** | 1604 | 1611 | 1620 | 1615 | 1595 | **1609** |
|  | s0396lre | 806 | 811 | 810 | 809 | 809 | **809** | 1170 | 1163 | 1181 | 1166 | 1157 | **1167** |
| patient94 | s0368lre | 1229 | 1231 | 1230 | 1231 | 1233 | **1231** | 1579 | 1584 | 1571 | 1579 | 1572 | **1577** |
|  | s0370lre | 589 | 596 | 590 | 594 | 594 | **593** | 861 | 890 | 875 | 861 | 856 | **869** |
|  | s0376lre | 851 | 856 | 856 | 854 | 847 | **853** | 1172 | 1190 | 1178 | 1166 | 1215 | **1184** |
|  | s0412lre | 653 | 655 | 647 | 655 | 653 | **653** | 974 | 980 | 991 | 980 | 980 | **981** |
| patient95 | s0369lre | 1333 | 1326 | 1336 | 1333 | 1333 | **1332** | 1656 | 1670 | 1666 | 1651 | 1665 | **1662** |
|  | s0373lre | 1077 | 1082 | 1081 | 1080 | 1075 | **1079** | 1453 | 1453 | 1447 | 1455 | 1459 | **1453** |
|  | s0377lre | 1705 | 1709 | 1710 | 1712 | 1710 | **1709** | 2059 | 2053 | 2062 | 2044 | 2053 | **2054** |
|  | s0417lre | 1491 | 1493 | 1495 | 1500 | 1493 | **1494** | 1837 | 1830 | 1850 | 1828 | 1823 | **1834** |
| patient96 | s0379lre | 813 | 818 | 815 | 822 | 813 | **816** | 1129 | 1134 | 1130 | 1129 | 1114 | **1127** |
|  | s0381lre | 836 | 840 | 840 | 838 | 836 | **838** | 1184 | 1193 | 1192 | 1179 | 1177 | **1185** |
|  | s0385lre | 752 | 757 | 753 | 752 | 752 | **753** | 1148 | 1150 | 1151 | 1132 | 1125 | **1141** |
|  | s0395lre | 738 | 743 | 737 | 738 | 736 | **738** | 1120 | 1125 | 1135 | 1111 | 1123 | **1123** |
| patient97 | s0380lre | 630 | 628 | 636 | 639 | 640 | **635** | 983 | 1025 | 1002 | 1002 | 992 | **1001** |
|  | s0382lre | 1204 | 1193 | 1203 | 1203 | 1204 | **1201** | 1584 | 1588 | 1588 | 1572 | 1572 | **1581** |
|  | s0384lre | 2935 | 2920 | 2929 | 2935 | 2935 | **2931** | 3387 | 3351 | 3373 | 3346 | 3356 | **3363** |
|  | s0394lre | 1109 | 1098 | 1105 | 1102 | 1107 | **1104** | 1477 | 1482 | 1485 | 1466 | 1471 | **1476** |
| patient98 | s0386lre | 1150 | 1154 | 1151 | 1150 | 1145 | **1150** | 1498 | 1491 | 1506 | 1487 | 1480 | **1492** |
|  | s0389lre | 1750 | 1752 | 1751 | 1758 | 1745 | **1751** | 2105 | 2114 | 2121 | 2098 | 2098 | **2107** |
|  | s0398lre | 1319 | 1324 | 1314 | 1319 | 1319 | **1319** | 1674 | 1679 | 1675 | 1667 | 1670 | **1673** |
|  | s0409lre | 1102 | 1102 | 1100 | 1107 | 1100 | **1102** | 1414 | 1425 | 1428 | 1412 | 1416 | **1419** |
| patient99 | s0387lre | 1014 | 1016 | 1018 | 1016 | 1014 | **1016** | 1344 | 1346 | 1349 | 1337 | 1349 | **1345** |
|  | s0388lre | 937 | 946 | 945 | 937 | 942 | **941** | 1261 | 1249 | 1279 | 1245 | 1256 | **1258** |
|  | s0397lre | 888 | 890 | 897 | 890 | 890 | **891** | 1272 | 1254 | 1281 | 1256 | 1256 | **1264** |
|  | s0419lre | 1290 | 1294 | 1298 | 1288 | 1294 | **1293** | 1656 | 1656 | 1663 | 1636 | 1656 | **1653** |
| patient100 | s0399lre | 1084 | 1091 | 1089 | 1084 | 1091 | **1088** | 1364 | 1369 | 1371 | 1353 | 1337 | **1359** |
|  | s0401lre | 664 | 664 | 672 | 666 | 671 | **667** | 1021 | 1023 | 1018 | 1028 | 989 | **1016** |
|  | s0407lre | 1109 | 1111 | 1113 | 1102 | 1111 | **1109** | 1421 | 1425 | 1411 | 1430 | 1412 | **1420** |
| patient101 | s0400lre | 976 | 980 | 983 | 974 | 974 | **977** | 1285 | 1292 | 1279 | 1290 | 1299 | **1289** |
|  | s0410lre | 1046 | 1055 | 1054 | 1046 | 1046 | **1049** | 1337 | 1331 | 1346 | 1367 | 1376 | **1351** |
|  | s0414lre | 1258 | 1263 | 1262 | 1256 | 1254 | **1259** | 1588 | 1590 | 1574 | 1586 | 1581 | **1584** |
| patient102 | s0416lre | 1152 | 1157 | 1157 | 1157 | 1157 | **1156** | 1557 | 1548 | 1555 | 1559 | 1552 | **1554** |
| patient103 | s0332lre | 833 | 840 | 840 | 840 | 840 | **839** | 1292 | 1283 | 1298 | 1285 | 1290 | **1290** |
| patient104 | s0306lre | 750 | 752 | 750 | 752 | 748 | **750** | 1134 | 1132 | 1146 | 1134 | 1132 | **1136** |
| patient105 | s0303lre | 705 | 709 | 704 | 702 | 702 | **704** | 1055 | 1062 | 1065 | 1053 | 1059 | **1059** |
| patient106 | s0030_re | 822 | 827 | 818 | 824 | 824 | **823** | 1218 | 1220 | 1224 | 1220 | 1218 | **1220** |
| patient107 | s0199_re | 1193 | 1195 | 1197 | 1195 | 1197 | **1195** | 1559 | 1563 | 1555 | 1552 | 1545 | **1555** |
| patient108 | s0013_re | 1073 | 1066 | 1073 | 1071 | 1073 | **1071** | 1482 | 1487 | 1490 | 1487 | 1480 | **1485** |
| patient109 | s0349lre | 2940 | 2933 | 2937 | 2944 | 2937 | **2938** | 3392 | 3410 | 3403 | 3392 | 3387 | **3397** |
| patient110 | s0003_re | 1380 | 1374 | 1382 | 1383 | 1380 | **1380** | 1785 | 1789 | 1799 | 1796 | 1789 | **1792** |
| patient111 | s0203_re | 1181 | 1186 | 1181 | 1186 | 1184 | **1184** | 1505 | 1514 | 1517 | 1507 | 1493 | **1507** |
| patient112 | s0169_re | 1755 | 1761 | 1761 | 1755 | 1762 | **1759** | 2213 | 2227 | 2211 | 2231 | 2233 | **2223** |
| patient113 | s0018cre | 639 | 648 | 639 | 641 | 640 | **641** | 976 | 978 | 983 | 978 | 971 | **977** |
|  | s0018lre | 1437 | 1445 | 1443 | 1437 | 1443 | **1441** | 1755 | 1767 | 1769 | 1758 | 1767 | **1763** |
| patient114 | s0012_re | 1267 | 1272 | 1268 | 1267 | 1272 | **1269** | 1679 | 1672 | 1680 | 1665 | 1649 | **1669** |
| patient115 | s0023_re | 1326 | 1322 | 1325 | 1328 | 1317 | **1324** | 1676 | 1679 | 1682 | 1670 | 1654 | **1672** |
| patient116 | s0302lre | 1134 | 1132 | 1138 | 1138 | 1136 | **1136** | 1538 | 1552 | 1550 | 1523 | 1537 | **1540** |
| patient117 | s0291lre | 1333 | 1324 | 1333 | 1335 | 1326 | **1330** | 1690 | 1674 | 1699 | 1685 | 1685 | **1687** |
|  | s0292lre | 1498 | 1489 | 1497 | 1500 | 1489 | **1495** | 1850 | 1862 | 1861 | 1847 | 1848 | **1854** |
| patient118 | s0183_re | 1111 | 1118 | 1108 | 1116 | 1118 | **1114** | 1509 | 1523 | 1514 | 1518 | 1534 | **1520** |
| patient119 | s0001_re | 1100 | 1098 | 1094 | 1100 | 1096 | **1098** | 1507 | 1500 | 1525 | 1475 | 1493 | **1500** |
| patient120 | s0331lre | 931 | 940 | 932 | 931 | 931 | **933** | 1312 | 1317 | 1346 | 1308 | 1308 | **1318** |
| patient121 | s0311lre | 1129 | 1132 | 1130 | 1127 | 1127 | **1129** | 1477 | 1487 | 1490 | 1491 | 1493 | **1488** |
| patient122 | s0312lre | 1618 | 1610 | 1612 | 1620 | 1615 | **1615** | 1998 | 2003 | 2002 | 1992 | 2005 | **2000** |
| patient123 | s0224_re | 1279 | 1276 | 1281 | 1279 | 1270 | **1277** | 1620 | 1624 | 1623 | 1606 | 1624 | **1619** |
| patient125 | s0006_re | 865 | 856 | 869 | 867 | 870 | **865** | 1186 | 1190 | 1200 | 1190 | 1188 | **1191** |
| patient126 | s0154_re | 1077 | 1073 | 1078 | 1078 | 1080 | **1077** | 1457 | 1468 | 1460 | 1459 | 1480 | **1465** |
| patient127 | s0342lre | 1435 | 1437 | 1447 | 1438 | 1446 | **1441** | 1860 | 1860 | 1865 | 1850 | 1865 | **1860** |
|  | s0383lre | 1044 | 1046 | 1048 | 1044 | 1044 | **1045** | 1487 | 1493 | 1498 | 1489 | 1498 | **1493** |
| patient128 | s0182_re | 1190 | 1193 | 1192 | 1193 | 1186 | **1191** | 1581 | 1588 | 1590 | 1566 | 1602 | **1585** |
| patient129 | s0189_re | 827 | 824 | 829 | 818 | 829 | **825** | 1188 | 1188 | 1195 | 1188 | 1199 | **1192** |
| patient130 | s0166_re | 2037 | 2037 | 2037 | 2044 | 2037 | **2038** | 2484 | 2491 | 2498 | 2473 | 2514 | **2492** |
| patient131 | s0273lre | 1007 | 1010 | 1010 | 1010 | 1010 | **1009** | 1324 | 1344 | 1325 | 1312 | 1331 | **1327** |
| patient133 | s0393lre | 1229 | 1229 | 1227 | 1233 | 1231 | **1230** | 1613 | 1622 | 1615 | 1602 | 1622 | **1615** |
| patient135 | s0334lre | 1475 | 1477 | 1476 | 1477 | 1473 | **1476** | 1932 | 1945 | 1948 | 1932 | 1938 | **1939** |
| patient136 | s0205_re | 1010 | 1001 | 1010 | 1014 | 1012 | **1009** | 1270 | 1279 | 1279 | 1274 | 1288 | **1278** |
| patient137 | s0392lre | 1629 | 1628 | 1628 | 1628 | 1629 | **1628** | 2035 | 2025 | 2043 | 2035 | 2023 | **2032** |
| patient138 | s0005_re | 1105 | 1096 | 1105 | 1109 | 1100 | **1103** | 1432 | 1435 | 1439 | 1437 | 1435 | **1436** |
| patient139 | s0223_re | 802 | 793 | 796 | 793 | 797 | **796** | 1138 | 1154 | 1149 | 1150 | 1145 | **1147** |
| patient140 | s0019_re | 1073 | 1075 | 1075 | 1077 | 1073 | **1075** | 1439 | 1444 | 1444 | 1448 | 1459 | **1447** |
| patient141 | s0307lre | 1389 | 1394 | 1390 | 1401 | 1394 | **1394** | 1778 | 1783 | 1783 | 1767 | 1816 | **1785** |
| patient142 | s0351lre | 1050 | 1053 | 1065 | 1059 | 1055 | **1056** | 1455 | 1480 | 1471 | 1448 | 1455 | **1462** |
| patient143 | s0333lre | 1331 | 1326 | 1333 | 1333 | 1324 | **1329** | 1712 | 1717 | 1710 | 1712 | 1744 | **1719** |
| patient144 | s0341lre | 1636 | 1630 | 1636 | 1644 | 1636 | **1636** | 2048 | 2053 | 2073 | 2046 | 2057 | **2055** |
| patient145 | s0201_re | 1416 | 1425 | 1422 | 1419 | 1414 | **1419** | 1884 | 1889 | 1886 | 1884 | 1871 | **1883** |
| patient146 | s0007_re | 2229 | 2229 | 2238 | 2233 | 2236 | **2233** | 2550 | 2558 | 2554 | 2550 | 2552 | **2553** |
| patient147 | s0211_re | 1968 | 1969 | 1972 | 1974 | 1969 | **1970** | 2385 | 2374 | 2382 | 2378 | 2355 | **2375** |
| patient148 | s0335lre | 1041 | 1044 | 1054 | 1046 | 1048 | **1047** | 1432 | 1423 | 1447 | 1430 | 1446 | **1436** |
| patient149 | s0202are | 1057 | 1059 | 1062 | 1057 | 1066 | **1060** | 1383 | 1387 | 1379 | 1383 | 1392 | **1385** |
|  | s0202bre | 876 | 883 | 873 | 876 | 876 | **877** | 1202 | 1211 | 1205 | 1193 | 1231 | **1208** |
| patient150 | s0287lre | 1263 | 1254 | 1262 | 1265 | 1254 | **1260** | 1636 | 1647 | 1658 | 1640 | 1642 | **1645** |
| patient151 | s0206_re | 1265 | 1261 | 1271 | 1265 | 1263 | **1265** | 1706 | 1710 | 1715 | 1701 | 1703 | **1707** |
| patient152 | s0004_re | 937 | 935 | 934 | 940 | 935 | **936** | 1328 | 1335 | 1338 | 1342 | 1340 | **1337** |
| patient153 | s0391lre | 1256 | 1261 | 1262 | 1258 | 1261 | **1260** | 1629 | 1633 | 1630 | 1629 | 1638 | **1632** |
| patient154 | s0170_re | 1786 | 1775 | 1785 | 1783 | 1783 | **1782** | 2170 | 2179 | 2181 | 2166 | 2195 | **2178** |
| patient155 | s0301lre | 768 | 766 | 772 | 766 | 768 | **768** | 1136 | 1143 | 1140 | 1132 | 1139 | **1138** |
| patient156 | s0299lre | 1150 | 1161 | 1154 | 1161 | 1161 | **1157** | 1496 | 1493 | 1506 | 1493 | 1498 | **1497** |
| patient157 | s0338lre | 1883 | 1892 | 1886 | 1884 | 1887 | **1886** | 2351 | 2364 | 2371 | 2346 | 2360 | **2358** |
| patient158 | s0294lre | 1089 | 1087 | 1094 | 1093 | 1098 | **1092** | 1523 | 1532 | 1533 | 1518 | 1525 | **1526** |
|  | s0295lre | 1588 | 1587 | 1590 | 1588 | 1593 | **1589** | 2030 | 2019 | 2029 | 2012 | 2010 | **2020** |
| patient159 | s0390lre | 1161 | 1161 | 1161 | 1161 | 1166 | **1162** | 1502 | 1505 | 1516 | 1491 | 1516 | **1506** |
| patient160 | s0222_re | 1367 | 1370 | 1379 | 1378 | 1368 | **1372** | 1792 | 1794 | 1802 | 1795 | 1787 | **1794** |
| patient162 | s0193_re | 1159 | 1163 | 1162 | 1161 | 1168 | **1163** | 1513 | 1508 | 1518 | 1533 | 1533 | **1521** |
| patient163 | s0034_re | 1236 | 1240 | 1241 | 1231 | 1238 | **1237** | 1584 | 1590 | 1585 | 1581 | 1579 | **1584** |
| patient164 | s0024are | 1543 | 1543 | 1555 | 1541 | 1554 | **1547** | 1955 | 1968 | 1970 | 1941 | 1963 | **1959** |
|  | s0024bre | 775 | 781 | 772 | 775 | 772 | **775** | 1181 | 1188 | 1200 | 1184 | 1188 | **1188** |
| patient165 | s0322lre | 1222 | 1220 | 1221 | 1224 | 1220 | **1221** | 1611 | 1620 | 1617 | 1599 | 1611 | **1612** |
|  | s0323lre | 1864 | 1861 | 1865 | 1866 | 1866 | **1864** | 2249 | 2256 | 2260 | 2247 | 2245 | **2251** |
| patient166 | s0275lre | 1633 | 1635 | 1631 | 1624 | 1636 | **1632** | 2037 | 2046 | 2051 | 2039 | 2041 | **2043** |
| patient167 | s0200_re | 1545 | 1542 | 1545 | 1548 | 1548 | **1546** | 1986 | 1953 | 1991 | 1979 | 1953 | **1972** |
| patient168 | s0032_re | 1084 | 1087 | 1092 | 1084 | 1091 | **1088** | 1471 | 1484 | 1501 | 1464 | 1459 | **1476** |
|  | s0033_re | 1493 | 1493 | 1495 | 1493 | 1493 | **1493** | 1854 | 1862 | 1869 | 1846 | 1850 | **1856** |
| patient169 | s0328lre | 1746 | 1745 | 1746 | 1746 | 1751 | **1747** | 2136 | 2143 | 2146 | 2134 | 2143 | **2140** |
|  | s0329lre | 1966 | 1969 | 1968 | 1969 | 1974 | **1969** | 2369 | 2369 | 2373 | 2362 | 2364 | **2367** |
| patient170 | s0274lre | 1057 | 1050 | 1062 | 1048 | 1053 | **1054** | 1403 | 1405 | 1409 | 1392 | 1412 | **1404** |
| patient171 | s0364lre | 1154 | 1154 | 1159 | 1157 | 1148 | **1154** | 1563 | 1570 | 1571 | 1557 | 1572 | **1567** |
| patient172 | s0304lre | 1471 | 1468 | 1474 | 1471 | 1468 | **1470** | 1873 | 1884 | 1888 | 1866 | 1877 | **1878** |
| patient173 | s0305lre | 1401 | 1399 | 1406 | 1403 | 1403 | **1402** | 1789 | 1789 | 1789 | 1783 | 1803 | **1791** |
| patient174 | s0300lre | 1322 | 1322 | 1323 | 1322 | 1317 | **1321** | 1688 | 1690 | 1691 | 1681 | 1685 | **1687** |
|  | s0324lre | 1559 | 1557 | 1560 | 1561 | 1561 | **1560** | 1945 | 1938 | 1956 | 1937 | 1950 | **1945** |
|  | s0325lre | 1655 | 1659 | 1653 | 1658 | 1658 | **1657** | 2050 | 2057 | 2059 | 2053 | 2053 | **2054** |
| patient175 | s0009_re | 1125 | 1116 | 1124 | 1125 | 1125 | **1123** | 1462 | 1473 | 1476 | 1464 | 1471 | **1469** |
| patient176 | s0188_re | 1184 | 1190 | 1186 | 1186 | 1188 | **1187** | 1536 | 1541 | 1539 | 1532 | 1536 | **1537** |
| patient177 | s0366lre | 1963 | 1955 | 1967 | 1967 | 1955 | **1961** | 2500 | 2505 | 2500 | 2491 | 2507 | **2501** |
| patient178 | s0011_re | 1435 | 1430 | 1436 | 1439 | 1435 | **1435** | 1846 | 1848 | 1856 | 1828 | 1839 | **1843** |
| patient179 | s0176_re | 1123 | 1120 | 1127 | 1116 | 1118 | **1121** | 1468 | 1496 | 1487 | 1462 | 1480 | **1479** |
| patient180 | s0374lre | 1242 | 1240 | 1246 | 1245 | 1245 | **1244** | 1593 | 1595 | 1598 | 1579 | 1606 | **1594** |
|  | s0475_re | 1541 | 1541 | 1547 | 1542 | 1543 | **1543** | 1884 | 1891 | 1897 | 1875 | 1891 | **1888** |
|  | s0476_re | 989 | 992 | 994 | 992 | 989 | **991** | 1333 | 1342 | 1334 | 1331 | 1340 | **1336** |
|  | s0477_re | 1606 | 1601 | 1612 | 1608 | 1604 | **1606** | 1957 | 1964 | 1972 | 1948 | 1966 | **1961** |
|  | s0490_re | 1776 | 1777 | 1785 | 1780 | 1776 | **1779** | 2134 | 2143 | 2143 | 2127 | 2138 | **2137** |
|  | s0545_re | 1001 | 1003 | 1008 | 1003 | 1001 | **1003** | 1333 | 1342 | 1344 | 1324 | 1346 | **1338** |
|  | s0561_re | 1749 | 1752 | 1753 | 1753 | 1746 | **1751** | 2118 | 2123 | 2127 | 2116 | 2129 | **2123** |
| patient181 | s0204are | 1953 | 1958 | 1954 | 1953 | 1955 | **1955** | 2310 | 2328 | 2344 | 2326 | 2337 | **2329** |
|  | s0204bre | 1075 | 1082 | 1077 | 1077 | 1075 | **1077** | 1428 | 1437 | 1441 | 1430 | 1430 | **1433** |
| patient182 | s0308lre | 1236 | 1230 | 1234 | 1229 | 1231 | **1232** | 1615 | 1606 | 1626 | 1609 | 1620 | **1615** |
| patient183 | s0175_re | 971 | 974 | 971 | 974 | 972 | **972** | 1471 | 1514 | 1490 | 1471 | 1471 | **1483** |
| patient184 | s0363lre | 1059 | 1057 | 1061 | 1062 | 1055 | **1059** | 1407 | 1412 | 1412 | 1403 | 1419 | **1411** |
| patient185 | s0336lre | 992 | 992 | 989 | 994 | 983 | **990** | 1403 | 1401 | 1404 | 1389 | 1403 | **1400** |
| patient186 | s0293lre | 813 | 806 | 804 | 813 | 811 | **809** | 1154 | 1161 | 1170 | 1157 | 1157 | **1160** |
| patient187 | s0207_re | 1170 | 1168 | 1176 | 1172 | 1177 | **1173** | 1505 | 1509 | 1506 | 1500 | 1529 | **1510** |
| patient188 | s0365lre | 1206 | 1206 | 1203 | 1213 | 1204 | **1206** | 1541 | 1552 | 1542 | 1534 | 1550 | **1544** |
| patient189 | s0309lre | 953 | 955 | 956 | 949 | 955 | **954** | 1385 | 1394 | 1395 | 1398 | 1367 | **1388** |
| patient190 | s0040_re | 1179 | 1172 | 1168 | 1175 | 1170 | **1173** | 1590 | 1602 | 1607 | 1584 | 1622 | **1601** |
|  | s0041_re | 1168 | 1159 | 1159 | 1159 | 1159 | **1161** | 1579 | 1588 | 1588 | 1568 | 1575 | **1580** |
| patient191 | s0340lre | 1579 | 1577 | 1580 | 1581 | 1579 | **1579** | 1968 | 1981 | 1991 | 1970 | 1986 | **1979** |
| patient192 | s0048_re | 1044 | 1048 | 1043 | 1053 | 1050 | **1048** | 1428 | 1444 | 1439 | 1423 | 1423 | **1431** |
| patient193 | s0008_re | 1694 | 1696 | 1696 | 1696 | 1700 | **1696** | 2213 | 2222 | 2224 | 2211 | 2215 | **2217** |
| patient194 | s0310lre | 1642 | 1641 | 1639 | 1647 | 1642 | **1642** | 2023 | 2016 | 2051 | 2023 | 2064 | **2035** |
| patient195 | s0337lre | 1046 | 1041 | 1043 | 1053 | 1044 | **1045** | 1450 | 1446 | 1453 | 1457 | 1457 | **1453** |
| patient196 | s0002_re | 1127 | 1127 | 1135 | 1132 | 1134 | **1131** | 1572 | 1579 | 1573 | 1575 | 1599 | **1580** |
| patient197 | s0350lre | 1358 | 1355 | 1363 | 1360 | 1353 | **1358** | 1841 | 1821 | 1853 | 1832 | 1832 | **1836** |
|  | s0403lre | 1891 | 1894 | 1899 | 1893 | 1896 | **1895** | 2362 | 2362 | 2376 | 2351 | 2380 | **2366** |
| patient198 | s0402lre | 1161 | 1159 | 1178 | 1163 | 1163 | **1165** | 1527 | 1529 | 1539 | 1516 | 1541 | **1530** |
|  | s0415lre | 1487 | 1489 | 1495 | 1489 | 1491 | **1490** | 1864 | 1864 | 1880 | 1846 | 1864 | **1864** |
| patient199 | s0404lre | 912 | 922 | 913 | 915 | 917 | **916** | 1317 | 1322 | 1333 | 1306 | 1315 | **1319** |
| patient200 | s0405lre | 1247 | 1242 | 1252 | 1247 | 1242 | **1246** | 1719 | 1724 | 1720 | 1712 | 1740 | **1723** |
| patient201 | s0420_re | 1450 | 1444 | 1457 | 1453 | 1453 | **1451** | 1848 | 1848 | 1856 | 1855 | 1859 | **1853** |
|  | s0423_re | 1453 | 1453 | 1455 | 1453 | 1450 | **1453** | 1810 | 1823 | 1818 | 1812 | 1821 | **1817** |
| patient202 | s0421_re | 831 | 829 | 830 | 833 | 833 | **831** | 1184 | 1186 | 1186 | 1181 | 1177 | **1183** |
|  | s0422_re | 1150 | 1148 | 1149 | 1150 | 1148 | **1149** | 1520 | 1534 | 1542 | 1523 | 1527 | **1529** |
| patient203 | s0424_re | 1299 | 1293 | 1300 | 1291 | 1299 | **1296** | 1706 | 1701 | 1715 | 1712 | 1710 | **1709** |
| patient204 | s0425_re | 1258 | 1258 | 1265 | 1258 | 1267 | **1261** | 1719 | 1724 | 1731 | 1737 | 1751 | **1732** |
| patient205 | s0426_re | 1568 | 1566 | 1569 | 1568 | 1566 | **1567** | 1961 | 1963 | 1961 | 1952 | 1971 | **1962** |
| patient206 | s0427_re | 1310 | 1312 | 1314 | 1310 | 1308 | **1311** | 1679 | 1685 | 1691 | 1672 | 1694 | **1684** |
| patient207 | s0428_re | 901 | 906 | 899 | 899 | 901 | **901** | 1322 | 1326 | 1333 | 1317 | 1283 | **1316** |
| patient208 | s0429_re | 933 | 922 | 929 | 933 | 926 | **929** | 1324 | 1319 | 1333 | 1319 | 1319 | **1323** |
|  | s0430_re | 1193 | 1184 | 1192 | 1199 | 1197 | **1193** | 1523 | 1525 | 1531 | 1525 | 1527 | **1526** |
| patient209 | s0431_re | 1021 | 1019 | 1024 | 1023 | 1019 | **1021** | 1459 | 1462 | 1460 | 1450 | 1464 | **1459** |
| patient210 | s0432_re | 919 | 922 | 918 | 922 | 926 | **921** | 1364 | 1283 | 1322 | 1331 | 1346 | **1329** |
| patient211 | s0433_re | 1114 | 1114 | 1113 | 1116 | 1118 | **1115** | 1432 | 1435 | 1436 | 1432 | 1439 | **1435** |
| patient212 | s0434_re | 1416 | 1423 | 1428 | 1423 | 1423 | **1423** | 1755 | 1769 | 1775 | 1751 | 1764 | **1763** |
| patient213 | s0435_re | 1046 | 1046 | 1054 | 1041 | 1046 | **1047** | 1392 | 1387 | 1403 | 1387 | 1394 | **1393** |
| patient214 | s0436_re | 922 | 919 | 924 | 926 | 924 | **923** | 1251 | 1256 | 1262 | 1245 | 1251 | **1253** |
| patient215 | s0437_re | 1258 | 1258 | 1265 | 1261 | 1261 | **1261** | 1676 | 1661 | 1678 | 1658 | 1642 | **1663** |
| patient216 | s0438_re | 1507 | 1505 | 1506 | 1509 | 1505 | **1506** | 1853 | 1830 | 1845 | 1850 | 1807 | **1837** |
| patient217 | s0439_re | 1059 | 1055 | 1059 | 1062 | 1055 | **1058** | 1468 | 1477 | 1479 | 1468 | 1511 | **1481** |
| patient218 | s0440_re | 720 | 730 | 724 | 719 | 724 | **723** | 1184 | 1177 | 1192 | 1170 | 1184 | **1181** |
| patient219 | s0441_re | 628 | 630 | 631 | 628 | 630 | **629** | 917 | 919 | 926 | 910 | 919 | **918** |
| patient220 | s0442_re | 1205 | 1204 | 1211 | 1204 | 1206 | **1206** | 1599 | 1584 | 1579 | 1606 | 1606 | **1595** |
| patient221 | s0443_re | 1596 | 1592 | 1601 | 1598 | 1595 | **1596** | 2010 | 2014 | 2021 | 2003 | 2019 | **2013** |
| patient222 | s0444_re | 885 | 883 | 894 | 888 | 883 | **887** | 1256 | 1261 | 1271 | 1256 | 1267 | **1262** |
| patient223 | s0445_re | 1195 | 1197 | 1200 | 1199 | 1197 | **1198** | 1561 | 1552 | 1574 | 1554 | 1575 | **1563** |
|  | s0446_re | 1430 | 1432 | 1436 | 1432 | 1428 | **1432** | 1803 | 1807 | 1813 | 1796 | 1812 | **1806** |
| patient224 | s0447_re | 874 | 872 | 875 | 876 | 872 | **874** | 1294 | 1301 | 1311 | 1292 | 1297 | **1299** |
| patient225 | s0448_re | 1148 | 1152 | 1154 | 1152 | 1161 | **1153** | 1570 | 1568 | 1569 | 1561 | 1550 | **1564** |
| patient226 | s0449_re | 1589 | 1592 | 1590 | 1588 | 1584 | **1589** | 2007 | 2001 | 2008 | 1998 | 2003 | **2003** |
| patient227 | s0450_re | 781 | 788 | 788 | 775 | 784 | **783** | 1125 | 1136 | 1138 | 1125 | 1141 | **1133** |
| patient228 | s0451_re | 1726 | 1725 | 1731 | 1728 | 1738 | **1730** | 2127 | 2134 | 2143 | 2141 | 2159 | **2141** |
| patient229 | s0452_re | 1683 | 1687 | 1691 | 1681 | 1683 | **1685** | 2118 | 2123 | 2119 | 2109 | 2114 | **2117** |
|  | s0453_re | 1846 | 1842 | 1853 | 1846 | 1850 | **1847** | 2272 | 2281 | 2276 | 2267 | 2283 | **2276** |
| patient230 | s0454_re | 1247 | 1249 | 1252 | 1247 | 1251 | **1249** | 1645 | 1647 | 1658 | 1642 | 1658 | **1650** |
| patient231 | s0455_re | 1654 | 1657 | 1663 | 1656 | 1658 | **1658** | 2019 | 2025 | 2021 | 2010 | 2021 | **2019** |
| patient232 | s0456_re | 998 | 1001 | 1010 | 1003 | 1005 | **1003** | 1389 | 1394 | 1403 | 1394 | 1412 | **1398** |
| patient233 | s0457_re | 1543 | 1538 | 1550 | 1538 | 1545 | **1543** | 1927 | 1948 | 1945 | 1929 | 1945 | **1939** |
|  | s0458_re | 1238 | 1242 | 1249 | 1238 | 1245 | **1242** | 1640 | 1649 | 1655 | 1638 | 1661 | **1649** |
|  | s0459_re | 1134 | 1132 | 1143 | 1136 | 1136 | **1136** | 1541 | 1545 | 1560 | 1538 | 1563 | **1549** |
|  | s0482_re | 1224 | 1222 | 1230 | 1224 | 1224 | **1225** | 1599 | 1604 | 1599 | 1595 | 1602 | **1600** |
|  | s0483_re | 1637 | 1632 | 1644 | 1636 | 1638 | **1637** | 2016 | 2014 | 2032 | 2010 | 2012 | **2017** |
| patient234 | s0460_re | 1518 | 1514 | 1523 | 1516 | 1520 | **1518** | 1945 | 1954 | 1956 | 1945 | 1950 | **1950** |
| patient235 | s0461_re | 1428 | 1430 | 1430 | 1430 | 1428 | **1429** | 1812 | 1814 | 1816 | 1801 | 1814 | **1811** |
| patient236 | s0462_re | 1120 | 1118 | 1119 | 1123 | 1120 | **1120** | 1543 | 1543 | 1547 | 1523 | 1532 | **1538** |
|  | s0463_re | 1340 | 1340 | 1341 | 1342 | 1346 | **1342** | 1762 | 1764 | 1769 | 1744 | 1760 | **1760** |
|  | s0464_re | 1482 | 1477 | 1485 | 1484 | 1483 | **1482** | 1900 | 1896 | 1900 | 1889 | 1900 | **1897** |
| patient237 | s0465_re | 1190 | 1188 | 1195 | 1193 | 1197 | **1193** | 1579 | 1588 | 1598 | 1579 | 1593 | **1587** |
| patient238 | s0466_re | 1222 | 1220 | 1230 | 1224 | 1222 | **1224** | 1588 | 1595 | 1598 | 1579 | 1588 | **1590** |
| patient239 | s0467_re | 1218 | 1220 | 1224 | 1220 | 1224 | **1221** | 1570 | 1575 | 1579 | 1561 | 1577 | **1572** |
| patient240 | s0468_re | 892 | 892 | 902 | 897 | 897 | **896** | 1251 | 1256 | 1254 | 1249 | 1256 | **1253** |
| patient241 | s0469_re | 1715 | 1714 | 1723 | 1715 | 1717 | **1717** | 2109 | 2116 | 2124 | 2102 | 2118 | **2114** |
|  | s0470_re | 1561 | 1563 | 1574 | 1563 | 1570 | **1566** | 1957 | 1959 | 1972 | 1957 | 1967 | **1962** |
| patient242 | s0471_re | 919 | 917 | 932 | 922 | 924 | **923** | 1272 | 1276 | 1298 | 1270 | 1274 | **1278** |
| patient243 | s0472_re | 1150 | 1154 | 1159 | 1152 | 1159 | **1155** | 1473 | 1477 | 1482 | 1468 | 1482 | **1476** |
| patient244 | s0473_re | 1254 | 1249 | 1260 | 1251 | 1249 | **1253** | 1604 | 1611 | 1636 | 1604 | 1606 | **1612** |
| patient245 | s0474_re | 1502 | 1502 | 1512 | 1507 | 1500 | **1505** | 1864 | 1868 | 1875 | 1859 | 1873 | **1868** |
|  | s0480_re | 1572 | 1572 | 1582 | 1574 | 1572 | **1574** | 1950 | 1945 | 1962 | 1945 | 1948 | **1950** |
| patient246 | s0478_re | 1935 | 1933 | 1934 | 1936 | 1933 | **1934** | 2324 | 2326 | 2338 | 2316 | 2344 | **2330** |
| patient247 | s0479_re | 1211 | 1218 | 1219 | 1218 | 1218 | **1217** | 1606 | 1611 | 1601 | 1593 | 1597 | **1602** |
| patient248 | s0481_re | 1064 | 1059 | 1067 | 1057 | 1059 | **1061** | 1446 | 1448 | 1460 | 1439 | 1446 | **1448** |
| patient249 | s0484_re | 1534 | 1532 | 1547 | 1538 | 1536 | **1537** | 1948 | 1952 | 1970 | 1941 | 1959 | **1954** |
| patient250 | s0485_re | 1261 | 1265 | 1271 | 1265 | 1265 | **1265** | 1674 | 1681 | 1688 | 1670 | 1685 | **1680** |
| patient251 | s0486_re | 1242 | 1242 | 1257 | 1245 | 1242 | **1246** | 1611 | 1624 | 1642 | 1610 | 1633 | **1624** |
|  | s0503_re | 1263 | 1263 | 1276 | 1265 | 1261 | **1266** | 1629 | 1638 | 1647 | 1629 | 1638 | **1636** |
|  | s0506_re | 935 | 935 | 945 | 937 | 933 | **937** | 1297 | 1303 | 1330 | 1299 | 1308 | **1307** |
| patient252 | s0487_re | 1871 | 1872 | 1875 | 1876 | 1874 | **1874** | 2272 | 2288 | 2292 | 2274 | 2292 | **2284** |
| patient253 | s0488_re | 1534 | 1533 | 1542 | 1538 | 1543 | **1538** | 1931 | 1935 | 1948 | 1936 | 1938 | **1938** |
| patient254 | s0489_re | 867 | 865 | 875 | 865 | 870 | **868** | 1177 | 1190 | 1181 | 1179 | 1186 | **1183** |
| patient255 | s0491_re | 908 | 910 | 918 | 910 | 919 | **913** | 1299 | 1299 | 1327 | 1299 | 1299 | **1305** |
| patient256 | s0492_re | 944 | 944 | 940 | 949 | 944 | **944** | 1389 | 1394 | 1406 | 1383 | 1374 | **1389** |
| patient257 | s0493_re | 1772 | 1770 | 1780 | 1778 | 1774 | **1775** | 2102 | 2134 | 2154 | 2129 | 2145 | **2133** |
| patient258 | s0494_re | 1211 | 1207 | 1208 | 1215 | 1206 | **1209** | 1586 | 1593 | 1609 | 1590 | 1602 | **1596** |
| patient259 | s0495_re | 1681 | 1675 | 1682 | 1676 | 1688 | **1680** | 2075 | 2084 | 2100 | 2071 | 2082 | **2082** |
| patient260 | s0496_re | 1602 | 1604 | 1602 | 1604 | 1602 | **1603** | 1954 | 1959 | 1959 | 1952 | 1957 | **1956** |
| patient261 | s0497_re | 1722 | 1732 | 1734 | 1724 | 1726 | **1728** | 2129 | 2136 | 2143 | 2129 | 2134 | **2134** |
| patient262 | s0498_re | 1683 | 1677 | 1688 | 1685 | 1683 | **1683** | 2129 | 2132 | 2151 | 2116 | 2116 | **2129** |
| patient263 | s0499_re | 1475 | 1468 | 1474 | 1473 | 1475 | **1473** | 1825 | 1828 | 1840 | 1823 | 1830 | **1829** |
| patient264 | s0500_re | 1387 | 1387 | 1390 | 1392 | 1387 | **1389** | 1792 | 1803 | 1793 | 1792 | 1803 | **1797** |
| patient265 | s0501_re | 1129 | 1125 | 1130 | 1125 | 1127 | **1127** | 1520 | 1525 | 1525 | 1507 | 1514 | **1518** |
| patient266 | s0502_re | 1294 | 1294 | 1303 | 1297 | 1306 | **1299** | 1663 | 1665 | 1674 | 1661 | 1679 | **1668** |
| patient267 | s0504_re | 1688 | 1687 | 1696 | 1690 | 1699 | **1692** | 2134 | 2138 | 2159 | 2133 | 2141 | **2141** |
| patient268 | s0505_re | 1010 | 1010 | 1010 | 1014 | 1014 | **1012** | 1346 | 1349 | 1346 | 1344 | 1349 | **1347** |
| patient269 | s0508_re | 1893 | 1890 | 1894 | 1896 | 1890 | **1893** | 2310 | 2312 | 2310 | 2301 | 2312 | **2309** |
| patient270 | s0507_re | 912 | 906 | 915 | 910 | 908 | **910** | 1324 | 1331 | 1336 | 1326 | 1324 | **1328** |
| patient271 | s0509_re | 1306 | 1306 | 1314 | 1308 | 1312 | **1309** | 1703 | 1708 | 1715 | 1701 | 1708 | **1707** |
| patient272 | s0510_re | 926 | 928 | 940 | 928 | 928 | **930** | 1285 | 1319 | 1322 | 1285 | 1317 | **1306** |
| patient273 | s0511_re | 1692 | 1693 | 1693 | 1685 | 1685 | **1690** | 2145 | 2175 | 2175 | 2145 | 2175 | **2163** |
| patient274 | s0512_re | 1724 | 1723 | 1734 | 1728 | 1726 | **1727** | 2136 | 2143 | 2159 | 2136 | 2111 | **2137** |
| patient275 | s0513_re | 761 | 759 | 764 | 763 | 766 | **763** | 1114 | 1123 | 1132 | 1116 | 1109 | **1119** |
| patient276 | s0526_re | 1089 | 1089 | 1092 | 1089 | 1091 | **1090** | 1453 | 1459 | 1490 | 1455 | 1473 | **1466** |
| patient277 | s0527_re | 1199 | 1202 | 1205 | 1195 | 1204 | **1201** | 1627 | 1636 | 1644 | 1640 | 1636 | **1637** |
| patient278 | s0528_re | 1202 | 1202 | 1205 | 1204 | 1202 | **1203** | 1552 | 1554 | 1552 | 1523 | 1559 | **1548** |
|  | s0529_re | 1141 | 1132 | 1140 | 1141 | 1141 | **1139** | 1489 | 1489 | 1489 | 1491 | 1493 | **1490** |
|  | s0530_re | 1091 | 1089 | 1092 | 1096 | 1096 | **1093** | 1437 | 1444 | 1441 | 1432 | 1428 | **1436** |
| patient279 | s0531_re | 2308 | 2309 | 2306 | 2310 | 2312 | **2309** | 2751 | 2759 | 2766 | 2749 | 2764 | **2758** |
|  | s0532_re | 2025 | 2021 | 2032 | 2025 | 2032 | **2027** | 2491 | 2516 | 2509 | 2489 | 2502 | **2501** |
|  | s0533_re | 2446 | 2442 | 2444 | 2445 | 2445 | **2444** | 2920 | 2927 | 2926 | 2906 | 2920 | **2920** |
|  | s0534_re | 2448 | 2455 | 2455 | 2455 | 2450 | **2453** | 2911 | 2932 | 2932 | 2932 | 2916 | **2925** |
| patient280 | s0535_re | 1780 | 1779 | 1788 | 1782 | 1780 | **1782** | 2168 | 2181 | 2197 | 2177 | 2177 | **2180** |
| patient281 | s0537_re | 1154 | 1154 | 1168 | 1154 | 1154 | **1157** | 1557 | 1561 | 1574 | 1552 | 1559 | **1561** |
| patient282 | s0539_re | 1658 | 1655 | 1669 | 1658 | 1663 | **1661** | 2037 | 2041 | 2078 | 2039 | 2053 | **2050** |
| patient283 | s0542_re | 1371 | 1371 | 1371 | 1374 | 1371 | **1372** | 1816 | 1823 | 1866 | 1823 | 1825 | **1831** |
| patient284 | s0543_re | 876 | 879 | 880 | 881 | 881 | **879** | 1240 | 1236 | 1260 | 1251 | 1236 | **1245** |
|  | s0551_re | 1100 | 1102 | 1100 | 1102 | 1100 | **1101** | 1457 | 1464 | 1471 | 1459 | 1475 | **1465** |
|  | s0552_re | 809 | 804 | 807 | 806 | 804 | **806** | 1161 | 1163 | 1189 | 1170 | 1170 | **1171** |
| patient285 | s0544_re | 0 | 0 | 0 | 0 | 0 | **0** | 0 | 0 | 0 | 0 | 0 | **0** |
| patient286 | s0546_re | 1025 | 1030 | 1043 | 1030 | 1032 | **1032** | 1435 | 1435 | 1433 | 1435 | 1433 | **1434** |
| patient287 | s0547_re | 1059 | 1050 | 1062 | 1059 | 1059 | **1058** | 1444 | 1428 | 1474 | 1446 | 1430 | **1444** |
|  | s0548_re | 725 | 727 | 731 | 729 | 725 | **727** | 1111 | 1107 | 1127 | 1145 | 1091 | **1116** |
| patient288 | s0549_re | 1612 | 1614 | 1620 | 1607 | 1615 | **1614** | 2050 | 2059 | 2075 | 2059 | 2050 | **2059** |
| patient289 | s0550_re | 1100 | 1101 | 1101 | 1101 | 1098 | **1100** | 1511 | 1493 | 1528 | 1493 | 1498 | **1505** |
| patient290 | s0553_re | 1825 | 1822 | 1823 | 1828 | 1825 | **1825** | 2254 | 2261 | 2284 | 2270 | 2276 | **2269** |
| patient291 | s0554_re | 809 | 811 | 815 | 818 | 820 | **815** | 1161 | 1193 | 1197 | 1143 | 1172 | **1173** |
| patient292 | s0555_re | 766 | 759 | 777 | 763 | 761 | **765** | 1098 | 1116 | 1113 | 1102 | 1123 | **1110** |
|  | s0556_re | 1258 | 1256 | 1265 | 1254 | 1254 | **1257** | 1599 | 1606 | 1620 | 1622 | 1606 | **1611** |
| patient293 | s0557_re | 802 | 804 | 815 | 804 | 809 | **807** | 1145 | 1190 | 1176 | 1186 | 1163 | **1172** |
|  | s0558_re | 1010 | 1001 | 1010 | 1012 | 1003 | **1007** | 1387 | 1367 | 1372 | 1397 | 1370 | **1379** |
| patient294 | s0559_re | 1077 | 1080 | 1086 | 1082 | 1077 | **1080** | 1441 | 1462 | 1471 | 1446 | 1462 | **1456** |
